# SUCNR1 coordinates metabolic flux, mitochondrial function, and nutrient-dependent adaptation in hepatocytes

**DOI:** 10.1126/sciadv.aec8873

**Published:** 2026-06-12

**Authors:** Anna Marsal-Beltran, Laura Salmerón-Pelado, Aleix Ribas-Latre, Maria Repollés-de-Dalmau, M-Mar Rodríguez-Peña, Catalina Núñez-Roa, Joan Badia, Ana Madeira, Eva Novoa, Marc Beltrà, Ana Belén Plata-Gómez, Jordi Capellades, Joan Carles Escolà-Gil, Antonio Zorzano, Óscar Yanes, Alejo Efeyan, Ruben Nogueiras, Jordi Gracia-Sancho, Joan Vendrell, Victòria Ceperuelo-Mallafré, Sonia Fernández-Veledo

**Affiliations:** ^1^ Institut de Recerca Biomèdica Catalunya Sud (formerly Institut d’Investigació Sanitària Pere Virgili), Hospital Universitari Joan XXIII, Tarragona 43005, Spain.; ^2^ CIBER de Diabetes y Enfermedades Metabólicas Asociadas (CIBERDEM), Instituto de Salud Carlos III, Madrid 28029, Spain.; ^3^ Rovira i Virgili University, Reus 43201, Spain.; ^4^ Plataformes de Bioinformàtica i de Suport Estadístic, Institut de Recerca Biomèdica Catalunya Sud-CERCA, Tarragona 43005, Spain.; ^5^ Department of Physiology, Center for Research in Molecular Medicine and Chronic Diseases (CIMUS), University of Santiago de Compostela, Santiago de Compostela 15782, Spain.; ^6^ CIBER de Fisiopatología de la Obesidad y Nutrición (CIBEROBN), Instituto de Salud Carlos III, Madrid 28029, Spain.; ^7^ Institute for Research in Biomedicine (IRB Barcelona), Barcelona Institute of Science and Technology, Barcelona 08028, Spain.; ^8^ Departament de Bioquímica i Biomedicina Molecular, Facultat de Biologia, Universitat de Barcelona, Barcelona 08028, Spain.; ^9^ Metabolism and Cell Signaling Laboratory, Spanish National Cancer Research Centre (CNIO), Madrid 28029, Spain.; ^10^ Institut de Recerca Sant Pau, Barcelona 08041, Spain.; ^11^ Universitat Rovira i Virgili, Department of Electronic Engineering. Institut de Recerca Biomèdica Catalunya Sud, Tarragona 43007, Spain.; ^12^ Biomedical Research Network on Hepatic and Digestive Diseases (CIBEREHD), Instituto de Salud Carlos III, Madrid 28029, Spain.; ^13^ Liver Vascular Biology, IDIBAPS Biomedical Research Institute, Barcelona 08036, Spain.; ^14^ Department of Visceral Surgery and Medicine, Inselspital, Bern University Hospital, University of Bern, Switzerland.

## Abstract

Succinate, a mitochondrial metabolite, also functions as an extracellular signal through its receptor succinate receptor 1 (SUCNR1), coordinating responses to nutrient availability. The physiological role of SUCNR1 within hepatocytes, however, is unclear. We show that hepatic succinate levels and *Sucnr1* expression are dynamically regulated by nutritional status. Mice lacking *Sucnr1* in hepatocytes [Hep-*Sucnr1* knockout (KO)] exhibit a fasting-like phenotype characterized by enhanced gluconeogenesis, elevated amino acids, and impaired metabolic flexibility. Mechanistically, loss of *Sucnr1* compromises glucose-derived oxidative flux through the tricarboxylic acid cycle, increases reliance on glutamine-dependent anaplerosis, and induces mitochondrial stress adaptations. Upon refeeding, Hep-*Sucnr1* KO mice show blunted mammalian target of rapamycin activation, incomplete glycogen restoration, and an altered hepatic proteomic response. *Sucnr1* expression increases during liver maturation, is enriched in pericentral hepatocytes, and its loss is associated with functional reprogramming of pericentral metabolic functions without disruption of zonation. Together, our findings establish SUCNR1 as a critical regulator of hepatic metabolic adaptation, linking succinate signaling to mitochondrial flexibility and nutrient-dependent metabolic responses.

## Teaser

The receptor for succinate SUCNR1 maintains hepatic metabolic flexibility and mitochondrial health.

## INTRODUCTION

The liver plays a central role in maintaining systemic homeostasis by dynamically responding to fluctuations in nutrient availability. This function is facilitated by the spatial zonation of hepatocytes, which exhibit distinct metabolic profiles according to their differential exposure to oxygen, nutrients, and hormones along the porto-central axis (*1*). For instance, periportal hepatocytes are characterized by the preferential use of β-oxidation pathway to obtain energy, while pericentral hepatocytes are more glycolytic because of the drop in oxygen availability (*2*).

Succinate is a key intermediate of the tricarboxylic acid (TCA) cycle, functioning both as a metabolic substrate and as a signaling molecule. In addition to fueling mitochondrial respiration via complex II (CII), succinate can accumulate and be exported to the extracellular space, where it activates the G protein–coupled receptor succinate receptor 1 (SUCNR1 or GPR91) (*3*, *4*). Notably, SUCNR1 is expressed across a wide array of tissues, with cell- and tissue-specific functions (*4*), and is increasingly recognized as a potential mediator of host-microbiota interactions, as succinate is also produced by gut bacteria (*5*). Although the succinate-SUCNR1 axis has been traditionally linked to pathological contexts, including obesity (*6*), type 2 diabetes (*7*, *8*), and metabolic dysfunction–associated steatotic liver disease (MASLD) (*9*–*11*), among others, recent data demonstrated that the succinate levels in circulation are responsive to food intake (*12*), indicating that succinate may have a physiological role in the organism through the activation of SUCNR1 in metabolic tissues. SUCNR1 was reported to stimulate leptin production in adipocytes (*13*) and insulin secretion in β cells (*14*). Focusing on the liver, no data on healthy hepatocytes are available, but in the context of MASLD, SUCNR1 was reported to protect against steatosis and decreased glycogen storage in in vivo and in vitro models (*9*). Given its role in modulating metabolic pathways in multiple tissues and its reported protective effects on lipid and glycogen metabolism in hepatocytes under lipid-overload conditions (*9*), it is plausible that succinate, via SUCNR1, contributes to the regulation of hepatic metabolic function. In this study, we investigated the physiological relevance of the succinate-SUCNR1 axis in the liver and its regulation by nutritional status.

We show that circulating succinate increases postprandially in mice and is accompanied by elevated hepatic succinate levels. Also, succinate secretion by hepatocytes is enhanced in response to glucose and glutamine exposure, implicating hepatocytes as a physiologically relevant source of circulating succinate in the fed state. Moreover, *Sucnr1* expression is nutritionally regulated, which supports a physiological role for hepatocyte SUCNR1 in adapting to nutrient availability. Hepatocyte-specific *Sucnr1* deletion mimics an energy-deprived state, promoting gluconeogenesis, altering mitochondrial substrate utilization, and eliciting adaptive TCA cycle and mitochondrial responses. During the transition from fasting to feeding, Hep-*Sucnr1* knockout (KO) mice display impaired mammalian target of rapamycin (mTOR) activation, partial restoration of hepatic glycogen, and a markedly altered proteomic profile. Notably, *Sucnr1* expression rises throughout postnatal liver development and is concentrated in pericentral hepatocytes, with its loss leading to a selective reprogramming of pericentral metabolic pathways while maintaining overall liver zonation. These observations highlight SUCNR1 as a central mediator of hepatic metabolic flexibility, integrating succinate signaling with mitochondrial function and nutrient-responsive adaptation.

## RESULTS

### Hepatic succinate secretion and *Sucnr1* expression are regulated by nutritional status

**Fig. 1.**
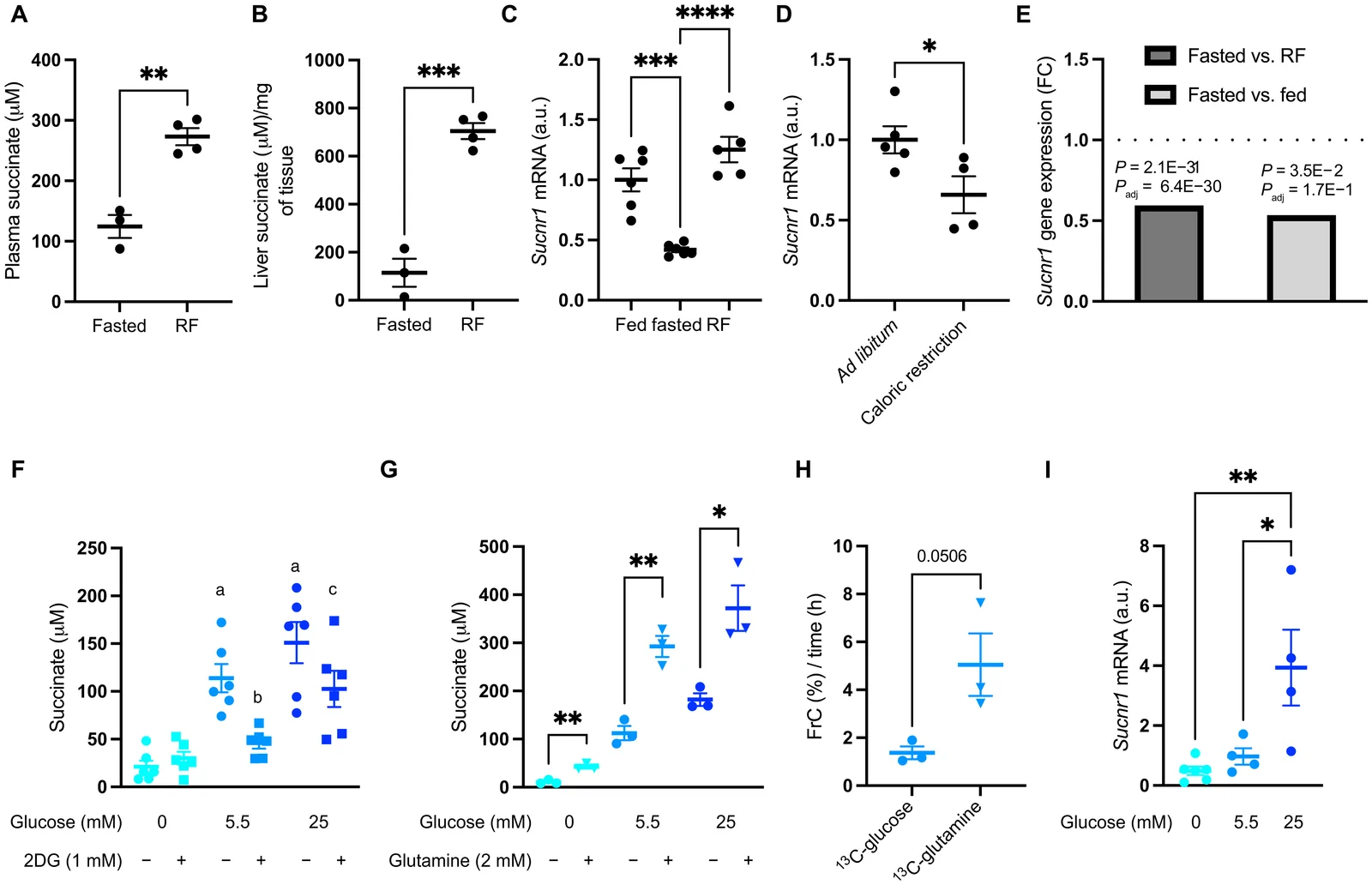
*Sucnr1* expression and succinate secretion in the liver are regulated in the fasted-to-fed transition. Plasma succinate in 8- to 12-week-old WT mice in overnight (o/n) fasting conditions or o/n fasting +2 hours (h) refed (RF) (*n* = 3 to 4) (**A**). Succinate in the livers of mice subjected to o/n fasting or o/n fasting +2 hours RF (*n* = 3 to 4) (**B**). *Sucnr1* mRNA expression in livers of 8- to 12-week-old WT mice ad libitum–fed, fasted for 24 hours, or fasted for 24 + 24 hours RF (*n* = 5 to 6) (**C**) and comparison between mice fed ad libitum or subjected to a caloric restriction (*n* = 5 to 4) (**D**). *Sucnr1* expression in murine livers extracted from transcriptomic data from two independent studies (*15*, *16*) comparing 24-hour fasting versus 24-hour fasting +2-hour RF (left bar) and 16-hour fasting versus control (right bar) (**E**). Succinate concentration in the conditioned medium of AML12 after 24-hour treatment of 0, 5, or 25 mM glucose, as well as 10 mM 2-deoxy-d-glucose (2DG) (*n* = 6) (**F**) or 2 mM glutamine (*n* = 3) (**G**). Fractional enrichment of succinate after administration of ^13^C-glucose or ^13^C-glutamine in primary hepatocytes of *Sucnr1*^fl/fl^ mice (*n* = 3) (**H**). *Sucnr1* mRNA expression after 24 hours of 0, 5, or 25 mM glucose treatment (*n* = 4 to 6) (**I**). Results are presented as means ± SEM [(A), (B), (C), (D), (F), (G), and (H)] or as fold change (fasted versus RF/fed) (E); *P < 0.05; **P < 0.01; ***P < 0.001; ****P < 0.0001; a, *P* < 0.05 versus 0 mM glucose w/o 2DG; b, *P* < 0.05 versus 5.5 mM glucose w/o 2DG; c, *P* < 0.05 versus 25 mM glucose w/o 2DG [two-tailed unpaired *t* test [(A), (B), (D), (G), and (H)]; one-way analysis of variance (ANOVA) plus Tukey’s multiple comparisons test [(C) and (I)]; one-way ANOVA plus Šídák’s multiple comparisons test (F). a.u., arbitrary units.

We initially investigated whether succinate and SUCNR1 are modulated by nutrient availability in the liver, with a particular focus on hepatocytes. Consistent with previous observations in humans (*12*), circulating succinate levels responded to nutritional changes, increasing in fed states compared to fasting animals (Fig. 1A). This systemic increase correlated with elevated succinate levels in the livers of 2-hour refed animals (Fig. 1B). Accordingly, we observed an up-regulation of *Sucnr1* in the livers of ad libitum–fed and 24-hour refed mice compared to those fasted for 24 hours (Fig. 1C). Similarly, a 60% caloric restriction for 4 days resulted in decreased *Sucnr1* gene expression (Fig. 1D). We further analyzed publicly available transcriptomic datasets from two independent studies (*15*, *16*), which confirmed our findings by showing a consistent ~50% reduction in *Sucnr1* expression in the liver during fasting (Fig. 1E).

In addition, plasma membrane succinate transporters, which mediate succinate uptake and release and are essential for its signaling functions, were also modulated by the nutritional status (fig. S1). At the cellular level, particularly in hepatocytes, glucose treatment stimulated succinate secretion, an effect decreased by coadministration of 2-deoxy-d-glucose, a nonmetabolizable glucose analog that competitively inhibits glycolysis (Fig. 1F). Succinate secretion by hepatocytes was also triggered by glutamine, which boosted the glucose effect (Fig. 1G). To further investigate the metabolic origin of secreted succinate, we performed stable isotope tracing experiments using ^13^C-labeled glucose and glutamine. Both substrates contributed to succinate labeling, indicating active carbon incorporation through glycolytic and glutaminolytic pathways. Notably, succinate ^13^C enrichment was higher following ^13^C-glutamine treatment in the presence of glucose, suggesting synergistic metabolic flux through anaplerotic entry points of the TCA cycle (Fig. 1H). The glucose-induced succinate secretion (Fig. 1F) correlated with an up-regulation of *Sucnr1* expression (Fig. 1I). These results suggest that the succinate/SUCNR1 axis in the liver may influence metabolic flexibility, positioning succinate as a potential metabolic regulator.

### Hepatic *Sucnr1* deficiency mimics energy deprivation and promotes gluconeogenesis

To further elucidate the role of SUCNR1 in hepatocytes, we generated a mouse model specifically lacking *Sucnr1* in hepatocytes by crossing mice with the floxed *Sucnr1* allele containing two loxP sites flanking exon 2 (*17*) with transgenic mice expressing Cre recombinase driven by the hepatocyte-specific *Alb* promoter. This resulted in *Sucnr1*^fl/fl^ *Alb-Cre*^+/−^ mice, hereafter referred to as Hep-*Sucnr1* KO mice. Cre-negative *Sucnr1*^fl/fl^ mice (*Sucnr1*^fl/fl^ *Alb-Cre*^−/−^) served as controls, referred to as *Sucnr1*^fl/fl^ mice (fig. S2). Hep-*Sucnr1* KO mice were born at a normal Mendelian ratio, were viable, and displayed no gross morphological differences between genotypes.

**Fig. 2.**
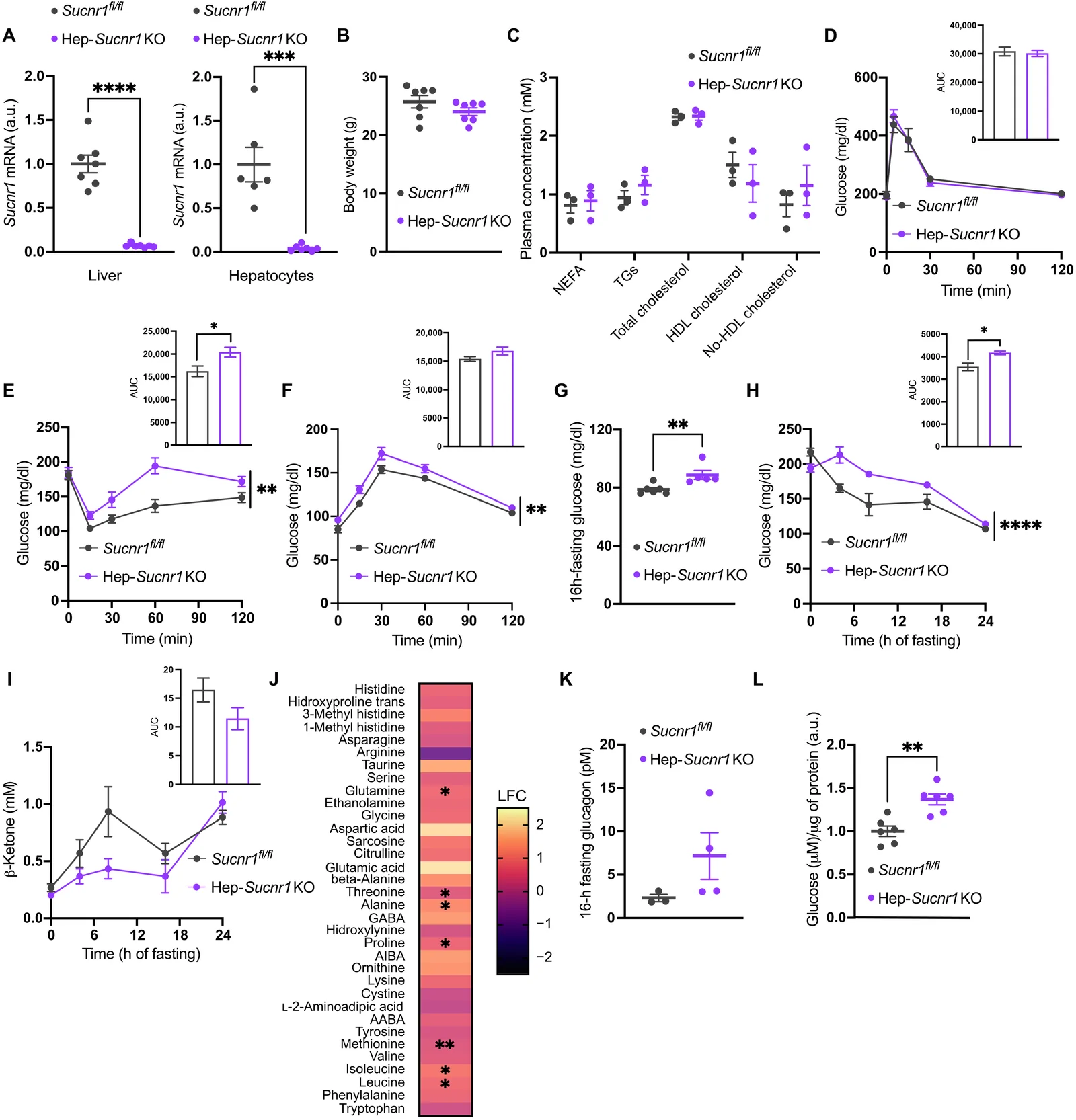
Mice lacking *Sucnr1* in hepatocytes show increased gluconeogenic capacity. Reverse transcription quantitative polymerase chain reaction (RT-qPCR) of *Sucnr1* in livers and hepatocytes from 10-week-old Hep-*Sucnr1* KO mice compared to controls (*n* = 7 to 7; 6 to 7) (**A**). Body weight (*n* = 7 to 7) (**B**). Plasma lipid profile (*n* = 3 to 3) (**C**). Glucose tolerance test and area under the curve (*n* = 6 to 3) (**D**). ITT and area under the curve (*n* = 9 to 5) (**E**). PTT and area under the curve (*n* = 9 to 9) (**F**). Glucose concentration in plasma after an o/n fasting (*n* = 6 to 5) (**G**). Glucose (**H**) and ketones (**I**) concentration in plasma in response to fasting and area under the curve (*n* = 3 to 8). Amino acids in plasma after an o/n fasting (*n* = 3 to 3) (**J**). Glucagon response in plasma to an o/n fasting (*n* = 3 to 4) (**K**). Glucose in the medium of primary hepatocytes isolated from *Sucnr1*^fl/fl^ mice and Hep-*Sucnr1* KO mice subjected to a medium without glucose supplemented with gluconeogenic precursors (*n* = 3 to 3) (**L**). Results are presented as means ± SEM or as log_2_ fold change Hep-*Sucnr1* KO versus *Sucnr1*^fl/fl^ mice (L) and statistical significance studied by two-tailed unpaired *t* test [(A), (C), (J), (K), and (L), bar graphs]; Mann-Whitney test [(B), (C), (G), and (J)]; two-way ANOVA [(D) to (F)] or mixed-effects model [(H) and (I)]; *P < 0.05; **P < 0.01; ***P < 0.001; ****P < 0.0001. AABA, α-aminobutyric acid; AIBA, α-aminoisobutyric acid; GABA, γ-aminobutyric acid; HDL, high-density lipoprotein; NEFA, nonesterified fatty acids; TGs, triglycerides.

As expected, *Sucnr1* expression was negligible in the liver and isolated primary hepatocytes (Fig. 2A) of Hep-*Sucnr1* KO mice compared to *Sucnr1*^fl/fl^ controls. No differences were observed in other tissues, such as subcutaneous, visceral, and brown adipose tissues (fig. S3A), confirming the specific deletion of *Sucnr1* in hepatocytes. In adult male mice (8 to 12 weeks of age), *Sucnr1*^fl/fl^ and Hep-*Sucnr1* KO mice exhibited similar body weight (Fig. 2B), lipid profile (Fig. 2C), and glucose tolerance (Fig. 2D). However, an insulin tolerance test (ITT) revealed an increased glucose excursion in Hep-*Sucnr1* KO mice (Fig. 2E), which was accompanied by a slight but significantly enhanced gluconeogenic response in the pyruvate tolerance test (PTT) (Fig. 2F). In addition, after overnight fasting, glucose levels in the Hep-*Sucnr1* KO mice were higher (Fig. 2G). Although less pronounced, female mice showed similar differences in glucose metabolism (fig. S4). In contrast to control mice, which exhibited a progressive decline in glucose levels during fasting, Hep-*Sucnr1* KO mice maintained more stable glucose levels, particularly in the early stages of fasting (Fig. 2H). This metabolic stability suggests enhanced gluconeogenesis and was accompanied by a tendency for a delayed rise in circulating ketone bodies (Fig. 2I), indicating a slower metabolic shift toward fat oxidation and ketogenesis. While plasma levels of lactate, pyruvate, and glycerol remained unchanged (fig. S3B), Hep-*Sucnr1* KO mice showed a significant increase in circulating levels of several amino acids known to serve as gluconeogenic precursors, including glutamine, threonine, alanine, proline, methionine, isoleucine, and leucine (Fig. 2J), as well as a tendency toward elevated glucagon levels (Fig. 2K), a principal hormonal driver of gluconeogenesis.

The potential role of SUCNR1 as a gluconeogenic inhibitor was confirmed in primary hepatocytes isolated from *Sucnr1*^fl/fl^ and Hep-*Sucnr1* KO mice. Despite no differences in the expression levels of key gluconeogenic enzymes (glucose-6-phosphatase, G6PASE; phosphoenolpyruvate carboxykinase 1, PCK1) or regulatory transcription factors (sirtuin 1, SIRT1; forkhead box O1, FOXO1) (fig. S3C), *Sucnr1*-deficient hepatocytes produced significantly more glucose when incubated in glucose-free medium supplemented with gluconeogenic precursors (Fig. 2L). These findings indicate that hepatic *Sucnr1* deficiency mimics an energy-deprived state, promoting gluconeogenesis and disrupting metabolic adaptation. This phenotype is characterized by increased circulating gluconeogenic amino acids, suggesting that enhanced hepatic glucose production may be substrate-driven rather than signaling dependent. Thus, SUCNR1 signaling appears to restrain hepatic gluconeogenic flux under fasting conditions, likely acting as a modulatory checkpoint to fine-tune glucose output and maintain energy homeostasis.

### Loss of *Sucnr1* impairs TCA cycle flux and elicits compensatory mitochondrial adaptations in hepatocytes

**Fig. 3.**
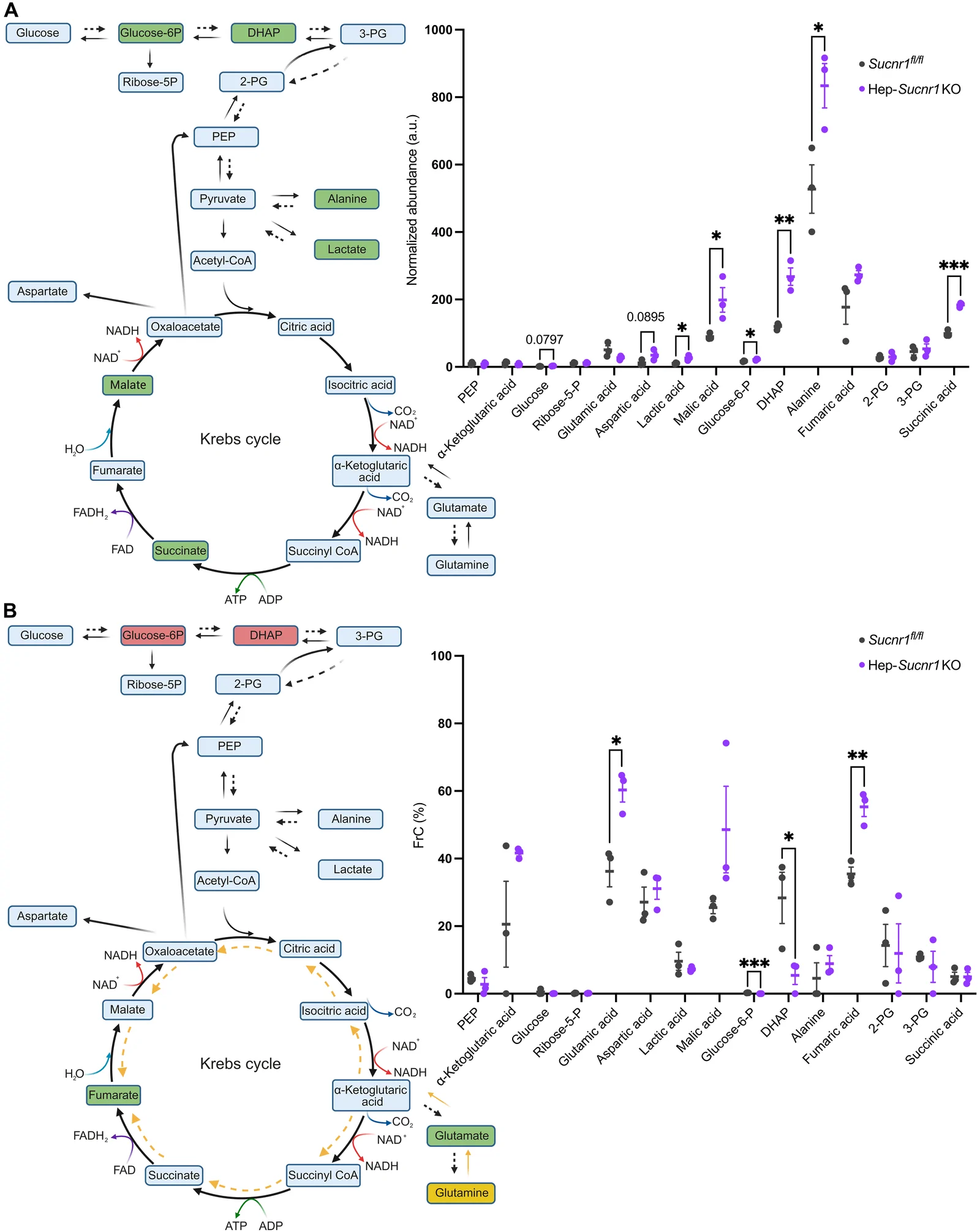
*Sucnr1* deletion regulates glutamine metabolism in hepatocytes. Normalized abundance (**A**) and fractional enrichment (**B**) of glycolytic, gluconeogenic, and TCA or Krebs cycle intermediates in response to ^13^C-glutamine administration to *Sucnr1*^fl/fl^ and Hep-*Sucnr1* KO hepatocytes for 1 hour (*n* = 3 to 3). Results are presented as means ± SEM and statistical significance studied by two-tailed unpaired *t* test or Mann-Whitney test; *P < 0.05; **P < 0.01; ***P < 0.001; green, up-regulated; red, down-regulated; yellow, suspected carbon flow. DHAP, dihydroxyacetone phosphate; glucose-6-P, glucose-6-phosphate; PEP, phosphoenolpyruvate; ribose-5-P, ribose-5-phosphate; 2-PG, 2-phosphoglycerate; 3-PG, 3-phosphoglycerate. Created in BioRender. Fernández-Veledo, S. (2026) https://BioRender.com/6yx74jq.

To further characterize the metabolic adaptations associated with *Sucnr1* deficiency, we conducted stable isotope–based flux analysis in primary hepatocytes to functionally assess changes in substrate utilization and pathway activity. When cells were treated with ^13^C-glucose, Hep-*Sucnr1* KO hepatocytes showed reduced ^13^C labeling of succinate, indicating impaired glucose oxidation via the TCA cycle (fig. S5). Nevertheless, when cells were cultured in glucose- and glutamine-rich medium, Hep-*Sucnr1* KO hepatocytes exhibited increased accumulation of glucose-6-phosphate, dihydroxyacetone phosphate, lactate, alanine, malate, and succinate (Fig. 3A), consistent with enhanced glycolytic flux, anaplerotic entry into the TCA cycle, and augmented amino acid catabolism.

Tracing experiments using ^13^C-labeled glutamine revealed a significant increase in the fractional enrichment of glutamic acid and fumarate, along with a concomitant reduction in the labeling of glucose-6-phosphate and dihydroxyacetone-phosphate (Fig. 3B). These results suggest that glutamine is increasingly redirected toward oxidative TCA cycle anaplerosis to compensate for diminished glucose oxidation, as well as toward the reductive carboxylation pathway, possibly reflecting a disrupted NAD^+^/NADH balance and mitochondrial adaptation (*18*). Together, these findings highlight a critical role for SUCNR1 in sustaining mitochondrial TCA cycle activity and substrate-dependent anaplerosis and underscore its importance in maintaining hepatic metabolic flexibility.

**Fig. 4.**
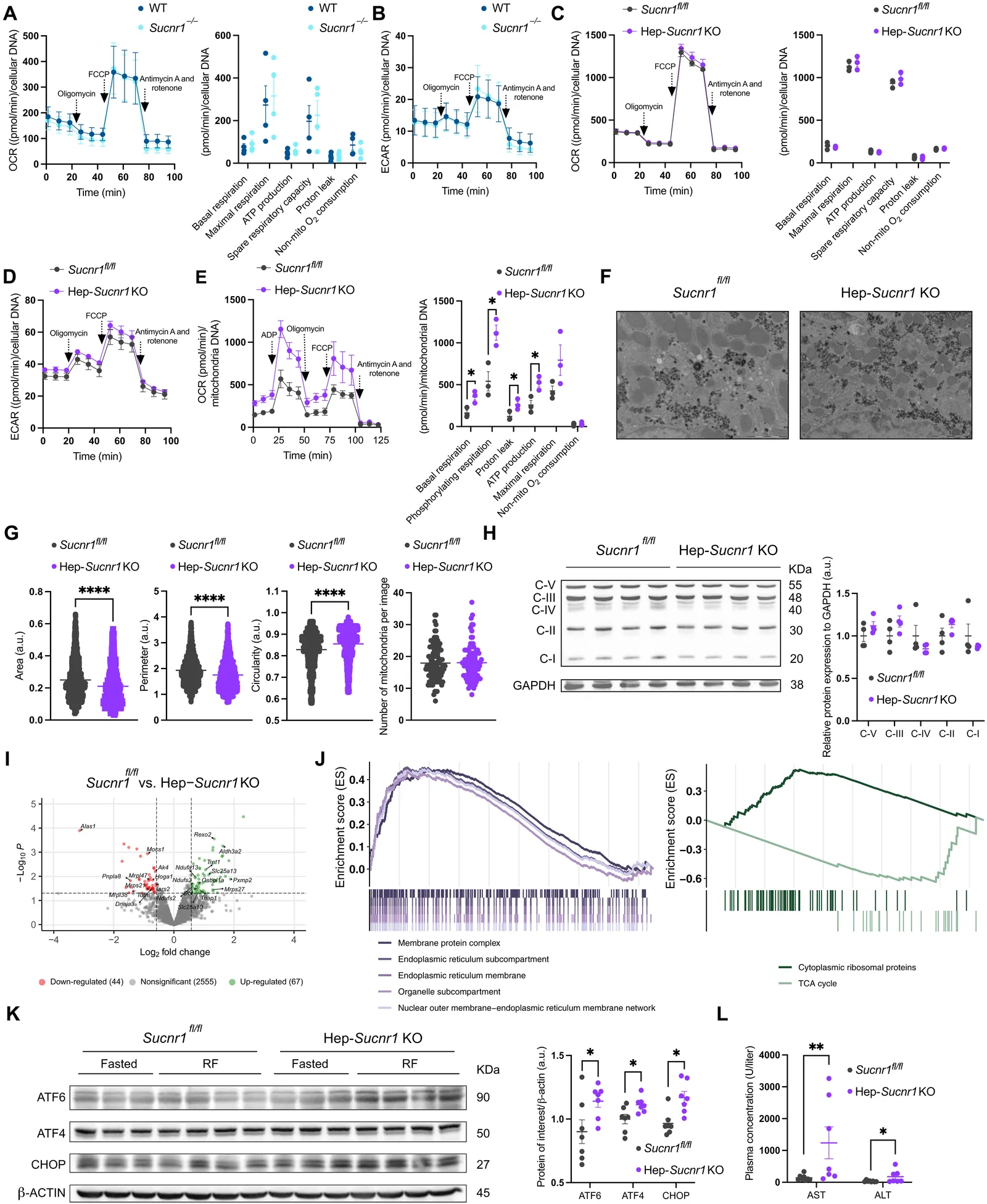
Lack of *Sucnr1* elicits compensatory mitochondrial adaptations and mild hepatic stress. OCR, basal and maximal respiration, ATP production, spare respiratory capacity, proton leak, and nonmitochondrial O_2_ consumption in primary hepatocytes from WT and *Sucnr1*^−/−^ mice; substrates: 10 mM glucose, 2 mM l-glutamine, 1 mM sodium pyruvate (*n* = 4 to 4) (**A**). ECAR under the same conditions (*n* = 4 to 4) (**B**). OCR parameters [as in (A)] and ECAR in primary hepatocytes isolated from *Sucnr1*^fl/fl^ and Hep-*Sucnr1* KO mice (*n* = 3 to 3) (**C** and **D**). OCR, basal respiration, phosphorylating respiration, proton leak, ATP production, maximal respiration, and nonmitochondrial O_2_ consumption in isolated mitochondria from *Sucnr1*^fl/fl^ and Hep-*Sucnr1* KO livers; substrates: 10 mM succinate, 2 μM rotenone (*n* = 3 to 3) (**E**). Transmission electron microscopy images of *Sucnr1*^fl/fl^ and Hep-*Sucnr1* KO livers (**F**). Area, perimeter, circularity, and number of mitochondria in the liver (*n* = 4 to 4) (**G**). Western blot of total OXPHOS complexes in liver extracts (*n* = 4 to 4) (**H**). Volcano plot of liver proteomics: Hep-*Sucnr1* KO versus *Sucnr1*^fl/fl^; labeled gene symbols denote mitochondrial proteins with *P* < 0.05 and −0.58 < log_2_ fold change <0.58 (*n* = 3 to 3) (**I**). Significantly enriched GO cellular component and Wiki pathways between genotypes (*n* = 3 to 3) (**J**). Immunoblot of ATF6, ATF4, and CHOP in o/n fasted (fasted) and o/n fasted plus 2 hours of refeeding (RF) livers, with densitometry normalized to β-actin (*n* = 7 to 7) (**K**). Plasma AST and ALT (*n* = 8 to 7) (**L**). Results are presented as means ± SEM; statistical analyses: two-tailed unpaired *t* test [(A), (B), (C), (D), (E), (H), and (K)] or Mann-Whitney test [(G), (H), (K), and (L)]. Proteomics are shown as log_2_ fold changes with limma’s empirical Bayes moderated *t* statistics (I). Pathway enrichment is presented as running enrichment scores (ES) across the ranked gene list (J); *P < 0.05; **P < 0.01; and ****P < 0.0001. ALT, alanine transaminase; AST, aspartate transaminase; ATF4, activating transcription factor 4; ATF6, activating transcription factor 6; C, complex; CHOP, C/EBP homologous protein.

To determine whether these alterations in substrate utilization and TCA cycle flux translate into functional changes in cellular energy metabolism, and given that succinate serves as a key indicator of mitochondrial bioenergetic state by linking TCA cycle activity to mitochondrial electron transport (*19*), we next assessed the role of SUCNR1 in mitochondrial respiration using Seahorse extracellular flux analysis. In murine AML12 hepatocytes, silencing *Sucnr1* via small interfering RNA (siRNA) (fig. S6A) led to a marked reduction in mitochondrial respiration when using substrates glucose, glutamine, and pyruvate, as evidenced by a decreased oxygen consumption rate (OCR) (fig. S6B). Concurrently, extracellular acidification rate (ECAR) was also reduced (fig. S6C), suggesting impaired glycolytic flux and overall reduced metabolic activity under these conditions. These findings were replicated in human THLE-2 cells treated with an SUCNR1 antagonist and were reversed by the SUCNR1 agonist *cis*-epoxysuccinic acid (fig. S6, D and E). The reduced mitochondrial respiration was accompanied by a decrease in the proportion of mitochondria with polarized membranes (fig. S6F), while the expression of oxidative phosphorylation (OXPHOS) subunits remained unchanged (fig. S6G). By contrast, Seahorse analysis of primary hepatocytes isolated from global *Sucnr1* KO (*Sucnr1*^−/−^) mice (Fig. 4, A and B) or Hep-*Sucnr1* KO mice (Fig. 4, C and D) revealed no significant differences in either the OCR or ECAR compared to wild-type (WT) or *Sucnr1*^fl/fl^ controls, respectively. This was accompanied by an elevated basal respiration, phosphorylating respiration, proton leak, and adenosine triphosphate (ATP) production in isolated liver mitochondria from Hep-*Sucnr1* KO mice supplied with succinate in the presence of rotenone, thereby providing electrons to CII and blocking CI, respectively (Fig. 4E). In contrast, respiration supported by CI substrates malate and pyruvate was unaltered across mitochondria from the different genotypes (fig. S6H). These results suggest that enhanced CII-supported respiratory capacity may represent an adaptive response to reduced TCA cycle flux, although this increase does not appear to be sufficient to markedly alter cellular respiration under substrate-limited conditions in vivo. Transmission electron microscopy of liver tissue from Hep-*Sucnr1* KO mice confirmed adaptive structural mitochondrial modifications, showing reduced mitochondrial area and perimeter, alongside increased circularity, and without changes in the number of mitochondria (Fig. 4, F and G), consistent with a fragmented or immature phenotype. These ultrastructural alterations occurred without differences in the expression of electron transport chain complexes (Fig. 4H), suggesting that enzyme abundance does not account for the observed differences in respiration of isolated mitochondria.

To further substantiate these findings, we performed quantitative liver proteomics under fasting conditions. We identified 147 significantly altered proteins in Hep-*Sucnr1* KO mice relative to controls (*P* < 0.05), 111 of which met both statistical significance and log_2_ fold change thresholds (−0.58 < × < 0.58) (Fig. 4I and table S1). Cross-referencing this dataset with the Mouse MitoCarta 3.0 database revealed that 24 of the dysregulated proteins are mitochondrially localized, which are named in Fig. 4I. Down-regulated proteins were primarily involved in mitochondrial protein translation (e.g., proteins encoded by *Tufm*, *Mrpl38*, *Mrps21*, and *Mrpl47* genes), heme biosynthesis (*Alas1*), phospholipid metabolism (Pnpla8), and energy homeostasis (*Ak4*), whereas up-regulated proteins included proteins with aldehyde dehydrogenase (NAD+) activity (*Aldh3a2*), components of CI (*Ndufa13*, *Ndufs2*, and *Ndufs3*), proteins related to amino acid metabolism (*Dbt*), metabolite transport and gluconeogenesis (*Slc25a10*), and regulation of mitochondrial transcription and apoptosis (e.g., *Rexo2* and *Triap1*), among others. Gene set enrichment analysis (GSEA) revealed a significant up-regulation of pathways related to endoplasmic reticulum (ER) stress, membrane complexes, and organelle interactions [Gene Ontology (GO) Cellular Component analysis], alongside a marked down-regulation of the TCA cycle components (Wiki pathways) (Fig. 4J), indicative of increased cellular stress and impaired mitochondrial metabolic function. Although no overt histological changes were detected (fig. S7), mediators of unfolded protein response (UPR) and integrated stress response (ISR) activating transcription factor 6 (ATF6), ATF4, and C/EBP homologous protein (CHOP), which were not responsive to refeeding, were elevated in Hep-*Sucnr1* KO livers under fasting plus refeeding conditions (Fig. 4K). Consistently, higher plasma aspartate transaminase (AST) and alanine transaminase (ALT) levels were observed in Hep-*Sucnr1* KO mice (Fig. 4L), suggesting subclinical liver injury likely related to fuel-limited mitochondria. These data identify SUCNR1 as a regulator of oxidative fluxes and show that its loss elicits mitochondrial adaptation, resulting in mild but measurable hepatic damage.

### *Sucnr1* deficiency disrupts hepatic protein response during metabolic transition from fasting to refeeding

**Fig. 5.**
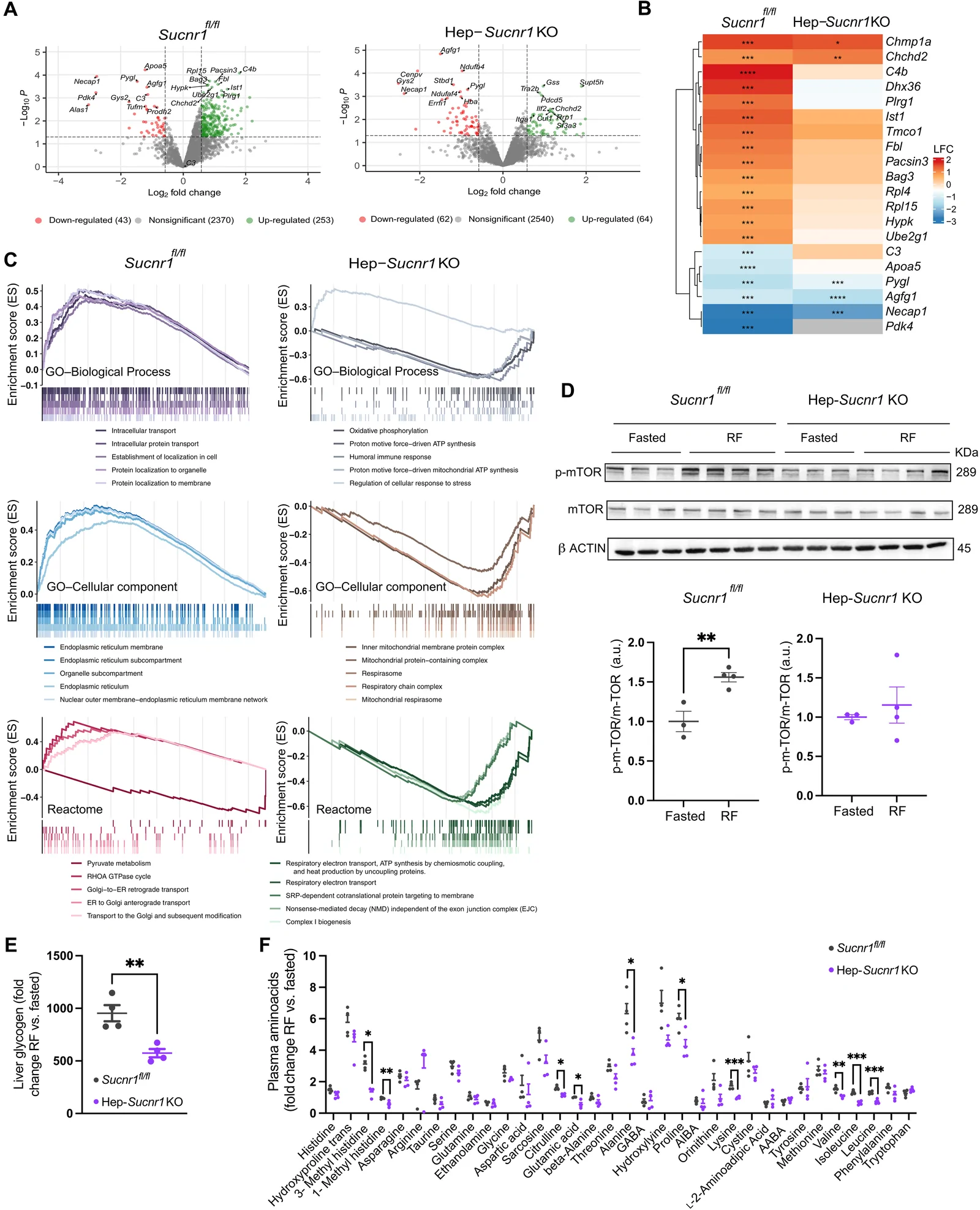
Lack of *Sucnr1* in hepatocytes reduces the hepatic refeeding response. Twelve-week-old *Sucnr1*^fl/fl^ and Hep-*Sucnr1* KO mice were subjected to o/n fasting (fasted) or o/n fasting +2 hours of refeeding (RF), and the livers and plasma were collected for analysis. Volcano plot illustrating the differential protein expression between RF and fasting in *Sucnr1*^fl/fl^ (left) and Hep-*Sucnr1* KO (right) mice with *P* value <0.05 and −0.58 < log_2_ fold change <0.58 (*n* = 3 to 4) (**A**). Log_2_ fold change (LFC) RF versus fasting in *Sucnr1*^fl/fl^ and Hep-*Sucnr1* KO of the top 20 most significantly different proteins in *Sucnr1*^fl/fl^ mice (*n* = 4 to 4) (**B**). GSEA for GO Biological Process, GO Cellular Component, and Reactome pathways in *Sucnr1*^fl/fl^ (top) and Hep-*Sucnr1* KO (bottom) mice (*n* = 3 to 4) (**C**). Western blot analysis of phosphorylated mTOR (p-mTOR) and total mTOR, with densitometric quantification of the p-mTOR/total mTOR ratio in livers of *Sucnr1*^fl/fl^ and Hep-*Sucnr1* KO mice in Fasted (*n* = 3) and RF (*n* = 4) conditions, normalized to β actin (**D**). Fold change of glycogen content RF versus fasting in the livers of *Sucnr1*^fl/fl^ and Hep-*Sucnr1* KO mice (*n* = 4 to 4) (**E**). Fold change of plasma amino acids RF versus fasting in *Sucnr1*^fl/fl^ and Hep-*Sucnr1* KO mice (*n* = 3 to 4) (**F**). Results are presented as log_2_ fold changes with limma’s empirical Bayes moderated *t* statistics [(A) and (B)] or as running enrichment score (ES) across the ranked gene list, with vertical bars marking the positions of pathway members (C). Results are shown as means ± SEM and statistical significance studied by two-tailed unpaired *t* test [(D) to (F)] or Mann-Whitney test [(D) and (F)]; *P < 0.05; **P < 0.01; ***P < 0.001. GABA, γ-aminobutyric acid; AIBA, α-aminoisobutyric acid; AABA, α-aminobutyric acid.

Given the link between mitochondrial dysfunction and reduced metabolic flexibility, we assessed the impact of *Sucnr1* deficiency on the hepatic adaptation to refeeding following fasting. Using a quantitative proteomics approach, we observed a marked impairment in the hepatic proteomic response of Hep-*Sucnr1* KO mice compared to controls after a 2-hour refeeding period. Specifically, while 475 proteins were significantly regulated in the control group, only 195 were differentially expressed in Hep-*Sucnr1* KO mice (table S1 and Fig. 5A). Among the top 20 proteins regulated by refeeding in controls, only five overlapped with those in the Hep-*Sucnr1* KO mice (encoded by *Chmp1a*, *Chchd2*, *Pygl*, *Agfg1*, *Necap1* genes; Fig. 5B), and the top 20 proteins identified in Hep-*Sucnr1* KO mice displayed a distinct pattern (Fig. 5B and fig. S8, A and B). Pathway enrichment analysis further revealed blunted activation of key pathways across GO categories (Biological Process and Cellular Component) and Reactome pathways. Although different pathways were modulated in each group, control mice exhibited mostly enriched pathways, such as intracellular transport and ER membrane–related processes. Conversely, in Hep-*Sucnr1* KO mice, refeeding led primarily to pathway down-regulation, including OXPHOS and inner mitochondrial membrane complex (Fig. 5C). The blunted refeeding-induced increase in binding immunoglobulin (IgG) protein and phosphorylated eukaryotic translation initiation factor 2 alpha (p-eIF2α)/total eIF2α ratio in Hep-*Sucnr1* KO livers supported the pathway enrichment in the proteomic analysis (fig. S8C). Consistent with these findings, the typical fasting-induced suppression of mTOR phosphorylation observed in the control mice was impaired in the Hep-*Sucnr1* KO mice (Fig. 5D), and the physiological refeeding response was attenuated, as evidenced by reduced hepatic glycogen accumulation (Fig. 5E) and altered circulating amino acid profiles (Fig. 5F). These findings indicate that SUCNR1 is essential for the hepatic adaptation to fasting-refeeding transitions, and its deficiency might compromise nutrient handling, leading to metabolic inflexibility.

### SUCNR1 is postnatally induced, enriched in pericentral hepatocytes, and regulates pericentral metabolism

**Fig. 6.**
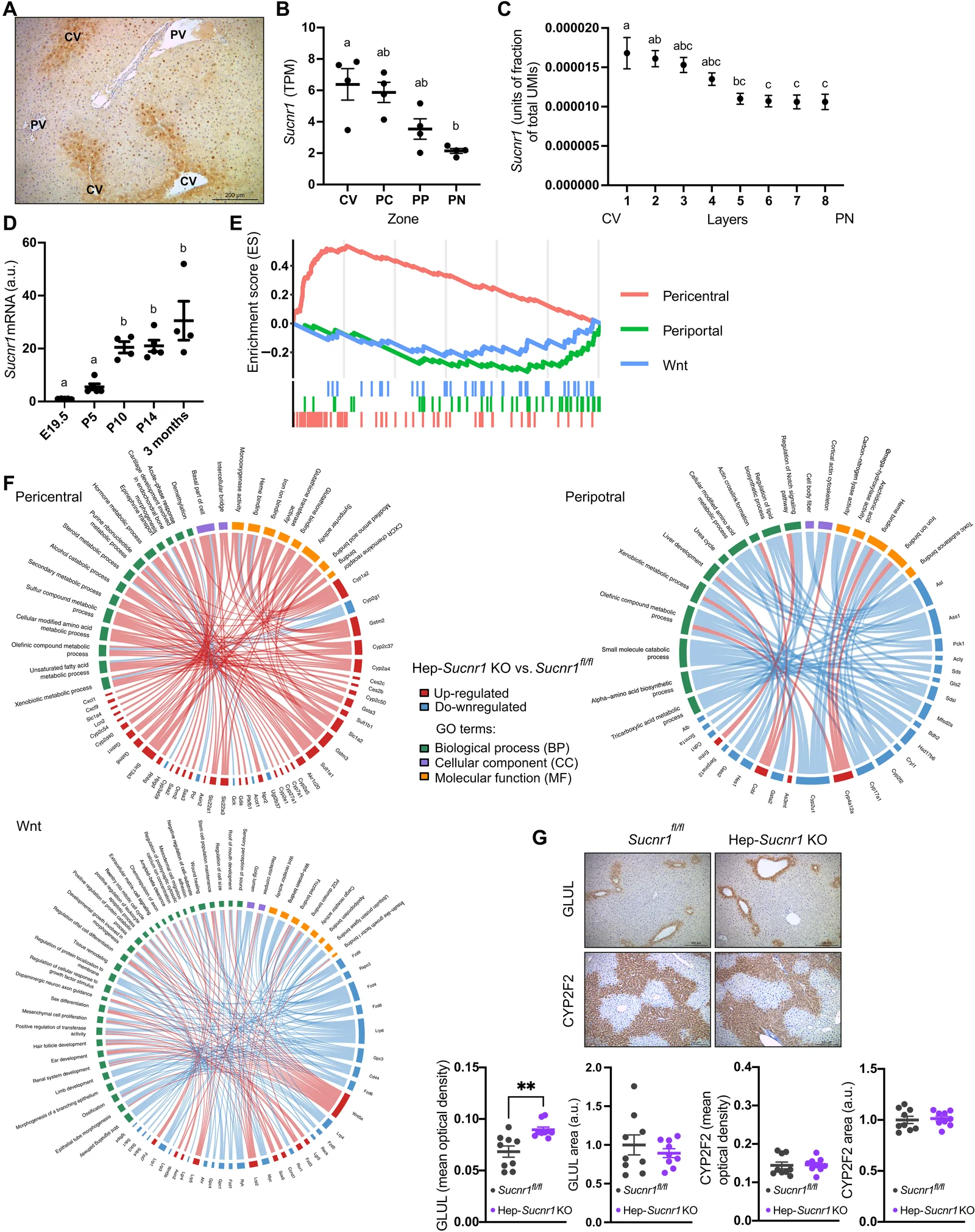
SUCNR1 is up-regulated in the liver during postnatal development, enriched pericentrally, and controls zonation-associated transcripts. SUCNR1 immunohistochemistry in adult WT mouse liver (**A**). *Sucnr1* expression (transcripts per million, TPM) in CD146 magnetic bead–prepurified LECs isolated from 30 C57BL/6 mice by fluorescence-activated cell sorting with c-kit antibody to separate four zones based on the gradient of expression along the porto-central axis of this protein (*21*): portal node (PN), periportal zone (PP), pericentral zone (PC), and central vein (CV) [data from table S1 in reference (*20*)] (**B**). *Sucnr1* expression [fraction of total unique molecular identifiers (UMIs)] in paired hepatocytes-LECs, isolated from three C57BL/6 mice assigned to layers one to eight according to zonation probability based on the expression of 21 pericentral and 30 periportal genes [data from table S5 in reference (*21*)] (**C**). RT-qPCR of *Sucnr1* mRNA in WT mouse liver at E19.5, P5, P10, and P14 (P5 to P14), and 3 months of age (*n* = 4 to 5) (**D**). GSEA of pericentral, periportal, and Wnt pathway gene signatures from hepatocytes’ transcriptomics data (Hep-*Sucnr1* KO mice versus *Sucnr1*^fl/fl^ mice) (*n* = 3 to 3) (**E**). Chord diagram of gene ontologies overrepresentation analysis in pericentral, periportal, and Wnt gene sets (*n* = 3 to 3) (**F**). Immunohistochemistry for glutamine synthetase (GLUL) and cytochrome P450 2F2 (CYP2F2) in *Sucnr1*^fl/fl^ and Hep-*Sucnr1* KO livers with quantification of mean optical density and stained area (*n* = 3 to 3) (**G**). Results are presented as means ± SEM; one-way ANOVA with Tukey’s multiple comparisons test (*P* < 0.05 indicated by different letters) [(B) to (D)]. GSEA results are shown as running enrichment scores (ES) across the ranked gene list (E). Results are shown as chord diagrams, with connection widths proportional to the absolute log_2_ fold change of each gene (F). Results are presented as means ± SEM and statistical significance studied by two-tailed unpaired *t* test or Mann-Whitney test (G); **P < 0.01.

Last, to determine the potential link between the succinate-SUCNR1 axis and the metabolic programming of hepatocytes across liver zones, we assessed SUCNR1 spatial and temporal expression in the liver. Immunohistochemical analysis of liver sections from control adult mice revealed that SUCNR1 was predominantly localized in pericentral hepatocytes (Fig. 6A). Although not specifically reported in the original studies, we interrogated publicly accessible supplementary datasets and identified a central expression pattern of *Sucnr1* in liver endothelial cells (LECs) isolated and profiled according to a c-Kit–based zonation protocol (Fig. 6B) (*20*, *21*) and in paired hepatocytes-LECs (Fig. 6C) (*21*). Since liver zonation is established during postnatal development, we examined *Sucnr1* expression at key developmental stages: embryonic day 19.5 (E19.5); postnatal days 5, 10, and 14 (P5, P10, and P14); and in 3-month-old adult mice. Our results showed a significant increase in *Sucnr1* mRNA expression during early postnatal development, with levels 20-fold higher at P10 and P14 compared to E19.5, and a 30-fold increase in adult mice (Fig. 6D). In addition, the expression of succinate transporters (*Slc13a3*, *Slc16a1*, and *Slc22a7*) also presented an early postnatal regulation (fig. S9).

Given that proteins regulated during postnatal development are known to orchestrate liver maturation and establish spatial hepatic identity (*22*), we investigated whether hepatic *Sucnr1* deficiency affects liver zonation. To this end, we conducted RNA sequencing (RNA-seq) analysis of primary hepatocytes isolated from *Sucnr1*^fl/fl^ and Hep-*Sucnr1* KO mice, focusing specifically on a curated set of liver zonation-associated genes and Wnt signaling components recently defined as key mediators of hepatic spatial patterning (full gene list in table S2) (*15*). Our transcriptomic analysis revealed a significant enrichment of pericentral gene expression signatures, whereas periportal markers and canonical Wnt signaling components were markedly down-regulated in *Sucnr1*-deficient hepatocytes (Fig. 6E and table S3). Specifically, GO overrepresentation analysis revealed distinct functional enrichments in the zonated gene sets. Up-regulated pericentral genes were associated to biological processes including cellular modified amino acid metabolism, hormone metabolism, and unsaturated fatty acid metabolism, suggesting roles in detoxification and complex molecule processing. By contrast, periportal genes were linked to TCA cycle metabolic processes, small molecules catabolism, and liver development, among others, reflecting a focus on energy production and biosynthesis. From the Wnt pathway gene set, genes with reduced expression clustered mainly in biological processes that included but were not limited to regulation of protein localization to the membrane, morphogenesis of a branching epithelium, and limb development, suggesting spatial regulation of developmental and signaling pathways within the liver (Fig. 6F). This transcriptional shift was accompanied by a trend toward increased circulating bile acids in Hep-*Sucnr1* KO mice (fig. S10), consistent with augmented bile acid synthesis, a process predominantly occurring in pericentral hepatocytes. To determine whether these modulations reflected alterations in hepatic zonation, classical zone 1 and zone 3 markers were examined through immunohistochemistry. No differences were found in the stained area for either glutamine synthetase (GLUL, pericentral) or cytochrome P450 2F2 (CYP2F2, periportal) (Fig. 6G). However, Hep-*Sucnr1* KO livers presented higher GLUL mean optical density without differences for CYP2F2 (Fig. 6G), indicating that loss of *Sucnr1* may selectively affect the metabolism of pericentral hepatocytes without disrupting overall liver zonation. Collectively, these findings suggest that SUCNR1 displays zonated expression in the liver and its absence induces compensatory up-regulation of pericentral functions, such as glutamine synthesis, while preserving liver spatial organization.

## DISCUSSION

Succinate is a key metabolite that, beyond its canonical role in mitochondrial energy metabolism, functions as an extracellular signaling molecule capable of orchestrating adaptive physiological responses in multiple organs, including adipose tissue (*13*) and the pancreas (*14*), through activation of its cognate receptor SUCNR1. Extending on this knowledge, this study assigns a direct physiological role to SUCNR1 specifically within hepatocytes. We identify SUCNR1 as an important modulator of hepatic metabolic adaptation, integrating nutrient availability with mitochondrial function and hepatocyte metabolic flexibility.

Our findings demonstrate that the postprandial rise in circulating succinate observed in humans (*12*) is recapitulated in mice, accompanied by increased hepatic succinate and its secretion by hepatocytes upon glucose and glutamine exposure. These results suggest hepatocytes as a relevant source of circulating succinate in the fed state. Moreover, *Sucnr1* expression was nutritionally regulated, increasing during refeeding and ad libitum feeding compared to fasting. Together with prior evidence from adipocytes (*13*), muscle (*23*), and β cells (*14*), these findings support a physiological role for SUCNR1 in hepatocytes in adapting to nutrient availability. Using a hepatocyte-specific *Sucnr1* KO model, we demonstrate that loss of *Sucnr1* results in a metabolic phenotype resembling prolonged energy deprivation. Hep-*Sucnr1* KO mice exhibit enhanced gluconeogenesis, elevated circulating gluconeogenic amino acids, and an impaired metabolic response to refeeding, despite preserved body weight and lipid profiles. These findings suggest that SUCNR1 signaling normally acts to restrain hepatic glucose production and facilitate the metabolic transition from fasting to feeding, thereby contributing to systemic glucose homeostasis.

At the cellular level, our isotope-tracing results demonstrate restricted glucose-derived oxidative flux through the TCA cycle, accompanied by a compensatory reliance on glutamine anaplerosis. These findings align with previous observations of metabolic rewiring under impaired oxidative conditions, such as those associated with mitochondrial DNA mutations (*24*), as well as in highly proliferative cells (*25*, *26*). These studies support a shift toward glutamine-dependent anaplerotic pathways when canonical oxidative glucose metabolism is constrained. Furthermore, this metabolic rewiring is accompanied by the accumulation of glycolytic and TCA cycle intermediates under nutrient-rich conditions, indicating impaired coupling between substrate availability and oxidative metabolism. Together, these data support a model in which SUCNR1 facilitates efficient utilization of glucose-derived carbon for mitochondrial oxidation, thereby sustaining metabolic flexibility.

Consistent with these findings, silencing or pharmacological inhibition of SUCNR1 in murine and human hepatocyte cell lines results in a marked reduction in mitochondrial respiration and membrane polarization, independent of changes in electron transport chain subunit abundance. These pronounced effects likely reflect the high metabolic demand and limited metabolic plasticity of proliferative cell lines, which rely heavily on continuous substrate-driven mitochondrial activity and lack the adaptive buffering mechanisms present in differentiated hepatocytes in vivo (*26*–*28*). By contrast, mitochondrial respiration in primary hepatocytes isolated from global or hepatocyte-specific *Sucnr1* KO mice is preserved, underscoring the importance of cellular context, differentiation state, and substrate availability in shaping the metabolic consequences of SUCNR1 loss. Notably, isolated liver mitochondria from Hep-*Sucnr1* KO mice show enhanced CII-supported respiration, while respiration with CI substrates (malate and pyruvate) is unchanged. This indicates an adaptive increase in succinate-driven electron flow, specifically compensating for reduced TCA cycle flux upstream rather than a global increase in mitochondrial capacity. Unlike intact cells, isolated mitochondria are supplied with saturating substrates and are uncoupled from cytosolic regulation, meaning that this enhanced respiratory capacity reflects intrinsic mitochondrial adaptations rather than physiological respiration in vivo (*29*). Accordingly, previous evidence links SUCNR1 to mitochondrial function (*30*–*34*), but inconsistent findings support a model in which SUCNR1-mediated mitochondrial regulation is tissue-, context-, and substrate-dependent. Collectively, our findings indicate that SUCNR1 modulates cellular energy demand and metabolic state in hepatocytes rather than directly controlling mitochondrial oxidative capacity, with mitochondrial adaptations becoming evident only when cellular-level regulation is removed.

In line with this interpretation, proteomic and ultrastructural analyses reveal evidence of mitochondrial remodeling and activation of cellular stress pathways in Hep-*Sucnr1* KO livers. We observe altered expression of mitochondrial proteins involved in translation, heme biosynthesis, and redox metabolism (*35*, *36*), accompanied by changes in mitochondrial morphology consistent with a fragmented or immature phenotype. Moreover, increased expression of UPR and ISR mediators, including ATF4, ATF6, and CHOP, together with elevated circulating transaminases, indicates mild but chronic hepatic stress (*37*). Accordingly, recent data from human liver biopsies have demonstrated an association between smaller and more rounded cytoplasmic mitochondria with liver enzymes and sensitivity to stress (*38*). These data suggest that SUCNR1 contributes to maintaining mitochondrial homeostasis under physiological nutrient fluctuations, limiting cellular stress associated with inefficient oxidative metabolism.

The impaired metabolic flexibility observed in Hep-*Sucnr1* KO mice becomes particularly evident during the transition from fasting to refeeding. While control livers mount a robust proteomic response to refeeding, characterized by activation of anabolic and organelle-associated pathways, this response is markedly blunted in the absence of *Sucnr1*. In our hepatocyte-specific KO model, we observe reduced mTOR activation, which has been associated with mitochondrial function (*39*) and incomplete glycogen restoration (*40*), aligning with our findings. In addition, altered amino acid handling further supports an affected hepatic nutrient response (*41*–*43*). Together with the divergence in pathway enrichment upon refeeding, these results support a role for SUCNR1 in coordinating nutrient-induced anabolic signaling with mitochondrial engagement during refeeding.

Last, our data indicate that the succinate-SUCNR1 axis exhibits a spatial dimension within the liver lobule. SUCNR1 expression increases during postnatal liver maturation and is enriched in pericentral hepatocytes in the adult liver, as supported by immunohistochemical analyses and reexamination of publicly available transcriptomic datasets (*20*, *21*). This expression pattern coincides with the establishment of hepatic metabolic specialization (*22*), suggesting that SUCNR1 signaling becomes particularly relevant in the mature liver. Specifically, it is enriched in the first layers of hepatocytes surrounding the central vein, corresponding to a distinct hepatocyte subpopulation within the pericentral zone (*44*), characterized by reduced gluconeogenic activity and increased engagement in glycolytic and glutamine-dependent pathways (*1*). In this context, the pericentral expression of SUCNR1 is consistent with a role in restraining gluconeogenesis, in line with the enhanced hepatic glucose production observed upon hepatocyte-specific deletion of *Sucnr1*. In addition, we examined the expression dynamics of the succinate transporter genes *Slc13a3* and *Slc22a7,* which are well-established as pericentral and periportal markers, respectively (*45*, *46*). Our data reveal that both genes are robustly expressed during early postnatal liver development but become markedly down-regulated in adulthood while remaining nutritionally responsive. These findings highlight the relevance of succinate transport and SUCNR1 signaling during postnatal metabolic reprogramming and suggest that this axis may also contribute to the hepatocyte’s adaptive response to nutritional cues, as the immediate postnatal period is marked by a major shift in active metabolic pathways (*16*, *22*).

Consistent with this view, transcriptomic analysis of isolated hepatocytes revealed that loss of *Sucnr1* is associated with modulation of zonation-related gene signatures, including up-regulation of pericentral metabolic genes and down-regulation of periportal markers and components of the Wnt signaling pathway. These changes were not accompanied by alterations in the spatial distribution of classical zonation markers such as GLUL or CYP2F2 (*47*), indicating that *Sucnr1* deletion does not disrupt overall liver zonal architecture but rather affects the functional state of hepatocytes within the pericentral zone. Together, these findings position SUCNR1 as a modulator of spatially defined metabolic functions within the liver lobule, linking succinate signaling to pericentral hepatocyte metabolism without altering the fundamental zonal organization of the tissue.

In conclusion, our study identifies SUCNR1 as a metabolic sensor that links succinate signaling to mitochondrial oxidative flux, cellular stress responses, and nutrient-dependent metabolic adaptation in hepatocytes. By facilitating efficient coupling between substrate availability and mitochondrial function, SUCNR1 supports hepatic metabolic flexibility during physiological fluctuations in nutrient supply. Dysregulation of this axis may therefore contribute to liver pathologies characterized by mitochondrial stress and impaired metabolic adaptability.

## MATERIALS AND METHODS

### Mice

#### Adult WT mice

For the fasting-refeeding experiment, 8- to 12-week-old WT mice (with a C57BL6J background) were subjected to ad libitum feeding, 24-hour fasting, or 24-hour fasting plus 24-hour refeeding, and the livers were harvested. For the caloric restriction experiment, the mice were individualized 5 days before the start of the experiments to monitor their daily food intake of ad libitum standard diet (6% kcal fat, 18% kcal protein, Teklad Global, Inotiv). Thereafter, the mice either continued with ad libitum feeding of standard diet or were subjected to a 60% caloric restriction, which consisted of each mouse being fed at 6:00 p.m. every day with an amount of food equal to 40% of their daily food intake during the period of acclimation. The mice were euthanized at 5.30 p.m. (before feeding) on the 4th day of caloric restriction to collect the liver for analyses. Animal experiments were conducted in accordance with the standards approved by the Faculty Animal Committee at the University of Santiago de Compostela (reference 15012/2023/014).

#### Postnatal development experiment

E19.5 livers were obtained after caesarean section at E19.5 (*48*) and placed on ice for euthanasia. Two milligrams of progesterone (P0130, Sigma-Aldrich) in phosphate-buffered saline (PBS) was injected to the pregnant mums once a day at E17.5 and E18.5 to prevent early delivery. Mice on P5, P10, P14, and P28 were euthanized in ad libitum conditions, and the livers were harvested for mRNA and histological analysis. All mouse procedures carried out at the CNIO were performed according to protocols approved by the CNIO-ISCIII Ethics Committee for Research and Animal Welfare (CEIyBA) and the Autonomous Community of Madrid, protocol numbers PROEX285/15, PROEX15/18 and PROEX225.7/22.

#### *Global Sucnr1 KO *(Sucnr1^−/−^)* mice*

Male C57BL/6J mice (WT) were purchased from The Jackson Laboratory, and *Sucnr1*^−/−^ mice on the same background were provided by K. McCreath (CNIC, Madrid, Spain) and have been previously described (*49*). Mice were maintained at the Faculty of Medicine and Health Science animal facility of Rovira i Virgili University and were housed three to six per cage under controlled conditions of 12-hour light/dark at 22°C with ad libitum access to chow diet (3.1% fat; SAFE diets, A04) and water. Animal studies were supervised and approved by the Universitat Rovira i Virgili Animal Welfare and Governmental Ethics Committee (reference 10970).

#### *Hep-*Sucnr1 *KO mice*

*Alb-Cre*^+/+^ mice with a C57BL6J background were kindly provided by Dr. Guadalupe Sabio (CNIC, Madrid, Spain) and were crossed to *Alb-Cre*^−/−^ *Sucnr1*^fl/fl^ mice to generate Hep-*Sucnr1* KO mice (*Alb-Cre*^+/−^ *Sucnr1*^fl/fl^). *Sucnr1*^fl/fl^ mice were used as controls (fig. S2). The two groups were maintained at the Faculty of Medicine and Health Science animal facility of Rovira i Virgili University and were housed three to six per cage under controlled conditions of 12-hour light/dark at 22°C with ad libitum access to chow diet (3.1% fat; SAFE diets, A04) and water unless otherwise stated. Body weight and food intake were recorded weekly. Before euthanasia, the mice were fasted overnight, followed by 2 hours of refeeding or continued with ad libitum access to food. The mice were then anesthetized with isoflurane, and blood samples were obtained by cardiac puncture. Blood samples, liver, and adipose tissues were harvested. Animal studies were supervised and approved by the Universitat Rovira i Virgili Animal Welfare and Governmental Ethics Committee (reference 10970).

All experimental procedures involving animals conformed to the European Union Directive 2010/63/EU and the European Commission Recommendation 2007/526/EC on the protection of animals used for experimental and other scientific purposes, enacted under the Spanish Royal Decrees 53/2013 and 118/2021.

### Metabolic assays

For the glucose tolerance test (GTT), mice underwent a 6-hour fast. Glucose (2 g/kg of body weight) was intraperitoneally injected, and blood glucose levels were monitored with a glucometer and glucose strips before the injection (time 0) and 15, 30, 60, 90, and 120 min after.

For the ITT, mice were starved for 3 hours before receiving an intraperitoneal infusion of human insulin (Actrapid) (0.75 U/kg of body weight in males and 0.60 U/kg of body weight in females). Glucose levels were determined before the injection and 15, 30, 60, 90, and 120 min after.

For the PTT, mice were fasted for 16 hours before receiving an intraperitoneal injection of sodium pyruvate (P2256-25G, Sigma-Aldrich) (1.5 g of sodium pyruvate/kg). Glucose levels were determined before the injection and 15, 30, 60, 90, and 120 min after. For the fasting studies, blood glucose and β-ketone levels were measured at different fasting time points in blood from the tail vein with a glucometer.

### Plasma lipid profile

Plasma total cholesterol (HICO GEN.2, 03039773190), triglycerides (ref. 20767107322), nonesterified fatty acids (NEFA-HR R1 and R2, 434-91795, 436-91995), ALT (20764957322), and AST (20764949322) were measured enzymatically and colorimetrically using commercial kits adapted for the COBAS C501/6000 autoanalyzer (Roche Diagnostics).

High-density lipoprotein (HDL) cholesterol levels were determined in plasma obtained after the precipitation of apolipoprotein B–containing lipoprotein particles using phosphotungstic acid (0.44 mM; Merck) and magnesium chloride (20 mM; Sigma-Aldrich), followed by enzymatic and colorimetric measurement using the cholesterol reagent adapted for the COBAS C501/6000 autoanalyzer. Very low–density plus low-density lipoprotein (VLDL+LDL) cholesterol was calculated as the difference between total plasma cholesterol and HDL cholesterol.

### Determination of pyruvate, lactate, glycerol, glucagon, and succinate in plasma or liver

Pyruvate, lactate, and glycerol in plasma samples were detected with the Pyruvate Assay Kit (MAK332, Sigma-Aldrich), the EnzyFluoTM l-lactate Assay Kit (EFLLC-100, BioAssay Systems), and the Glycerol Assay Kit (MAK117, Sigma-Aldrich), respectively. Plasma glucagon was evaluated with the Glucagon enzyme-linked immunosorbent assay −10 μl (10-1281-01, Mercodia). Plasma and liver succinate were determined with the EnzyChromTM Succinate Assay Kit (ESNT-100, BioAssay Systems).

### Bile acids determination by LC-MS/MS

Fifty microliters of plasma was mixed with 200 μl of acetonitrile containing internal standards (0.3 μM CA-d5, 0.1 conjugated mix), vortexed, and centrifuged for 10 min at 15000 rpm and 4°C. The supernatant was transferred to a new tube and evaporated in a SpeedVac at 45°C. Fifty microliters of methanol:water was used to reconstitute the samples, which were then transferred to glass vials for analysis. Chromatographic separation was performed on an ultrahigh-performance ultrahigh-performance LC (UHPLC) 1290 Infinity II Series coupled to a QqQ/MS 6490 Series (Agilent Technologies) with a Kinetex EVO C18 (150 mm by 2.1 mm) analytical column (Phenomenex). Mobile phase A was 0.1% ammonium hydroxide (Sigma-Aldrich) and 10 mM ammonium acetate (Sigma-Aldrich), and B was acetonitrile LC–mass spectrometry (LC-MS, Merck). The column temperature was set at 27°C, and the injection volume was 2 μl. Validation of the analytical methodology was carried out by analyzing both human plasma and additions to water mix using internal standards for each analyte.

### Determination of amino acids by LC-MS/MS

Ten microliters of plasma samples were mixed with 5 μl of MeOH containing standards (MSK-A2-S) and 35 μl of MeOH, vortexed, and centrifuged at 15,000 rpm and 4°C. The supernatant was transferred to a new tube and evaporated in a SpeedVac at 45°C. Fifty microliters of borate buffer was used to reconstitute the samples, and 10 μl were derivatized using AccQ-Tag reagent (Waters) following manufacturing protocol. Derivatized amino acids were analyzed by UHPLC–tandem MS (MS/MS) in multiple reaction monitoring acquisition. Chromatographic separation was performed on a UHPLC 1290 Infinity II Series coupled to a QqQ/MS 6490 Series (Agilent Technologies) with an ACQUITY UPLC HSS T3 Column, 100A, 1.8 μm, 2.1 mm by 100 mm (Waters Corporation). Mobile phase A was water with 0.1% formic acid, and B was acetonitrile with 0.1% formic acid. The column temperature was set at 40°C, and the injection volume was 2 μl.

### Liver H&E staining

The left liver lobe was washed in PBS, fixed in 4% paraformaldehyde, dehydrated, and paraffin-embedded. The samples were then sectioned, rehydrated, and stained with hematoxylin and eosin (H&E), following dehydration, mounting, and observation by light microscopy (Leica DM4B microscope) to examine hepatic steatosis. Images were acquired and processed with Leica Application Suite V4.13.

### SUCNR1, GLUL, and CYP2F2 immunohistochemistry in the liver

The left liver lobe was washed in PBS, fixed in 4% paraformaldehyde, dehydrated, and paraffin-embedded. The samples were then sectioned and rehydrated to continue with the immunohistochemistry protocol. The sections were heated to 95°C for 20 min in TE buffer (pH 9) for SUCNR1 and GLUL, or in citrate buffer (pH 6) for CYP2F2. Endogenous peroxidase activity was quenched by incubation with 0.3% H_2_O_2_ (216763, Sigma-Aldrich) for 30 min. The sections were blocked with 2% bovine serum albumin (BSA) (A7030, Sigma-Aldrich), 2% normal goat serum, and 0.2% fish gelatin (G7765, Sigma-Aldrich) in PBS for 30 min and subjected to a background removal process with F(ab’)2-goat anti-mouse IgG (H + L) secondary antibody (A24514, Invitrogen) for 1 hour in a humidified chamber. Primary antibody incubations were performed either overnight at 4°C with anti-SUCNR1 (1:500; ZRB2183, Sigma-Aldrich), overnight at 4°C with anti-glutamine synthetase (GLUL; 1:400; ab64613, Abcam), or for 1 hour at room temperature with anti-CYP2F2 (1:100; sc-374540, Santa Cruz Biotechnology), all diluted in PBS containing 1% BSA. Sections were subsequently incubated for 1 hour with horseradish peroxidase (HRP)–conjugated secondary antibodies: donkey anti-rabbit IgG (1:250; NA934V, ECL) for SUCNR1, or goat anti-mouse IgG (1:250; 62-6520, Invitrogen) for GLUL. Signal was developed using a DAB peroxidase substrate kit (SK-4100, Vector Laboratories). For CYP2F2 staining, the sections were incubated with biotinylated horse anti-mouse IgG (1:250; BA200, Vector Laboratories) for 15 min at room temperature, followed by signal amplification using the VECTASTAIN ABC peroxidase system (PK4000, Vector Laboratories) and detection with DAB substrate (SK-4100, Vector Laboratories). Mayer’s Hematoxylin (MHS32, Sigma-Aldrich) was used for counterstaining in all cases. After dehydration and mounting, the sections were observed in a Leica DM4B microscope and imaged with Leica Application Suite V4.13. Quantification of signal intensity and stained area for GLUL and CYP2F2 was performed using Fiji software in three images per mouse. For signal intensity analysis, color deconvolution was applied to isolate the DAB channel, and the mean gray value was measured and converted to mean optical density, proportional to DAB intensity. For stained area quantification, the DAB channel was isolated, and a default threshold was applied to calculate the percentage of positively stained area in each image.

### Liver proteomics

Liver tissue (20 mg) was lysed in Mammalian Protein Extraction Reagent (M-PER, 78501) supplemented with Halt Protease (1861278) and Phosphatase Inhibitor Cocktail (1861277) plus EDTA (1861275) (all from Thermo Fisher Scientific), and diluted to reach a 1 μg/μl concentration. After protein precipitation, the samples were digested with LysC and Trypsin and analyzed by LC-MS/MS using a 90-min gradient in the Orbitrap Eclipse by data-dependent acquisition. The samples were explored in the SwissProt Mouse database using the Mascot v2.6 algorithm. Peptides were filtered on the basis of false discovery rate (FDR), and only the ones with an FDR lower than 1% were retained. Data were normalized by total peptide amount. Differential protein abundance was determined via linear models with empirical Bayes moderation [R libraries: limma (version 3.60.6) (*50*); default settings]. *P* values lower than 0.05 were considered significant. Filters of a significant *P* value and a log_2_ fold change 0.58 < by < 0.58 (*51*) were applied to select a set of proteins differentially expressed between genotypes and search them in the Mouse MitoCarta 3.0 database (https://personal.broadinstitute.org/scalvo/MitoCarta3.0/mouse.mitocarta3.0.html), which is a collection of 1140 genes, both nuclear and mitochondrial DNA, encoding proteins with strong support of mitochondrial localization (*2*). Pathway analysis was conducted with GSEA with the following parameters: clusterProfiler (version 2.1.6) (*52*) and PathwayPA (version 1.48.0) (*53*) for R libraries; minimal gene set (column setSize) size to be included for analysis of 10 (GO Biological Process, GO Cellular Component) or 15 (WikiPathways, KEGG, Reactome); significance filter of *q* value <0.05; ranked by *q* values and, in case of exact *q* value, ranked by absolute normalized enrichment score value.

### Glycogen content determination in the liver

Intracellular glycogen levels were assessed using the Glycogen Assay Kit (ab65620, Biovision). Liver tissue (10 mg) was homogenized in deionized water on ice with a dounce homogenizer (2404F, GPE Scientific), followed by a 10-min boiling step to inactivate enzymes. The homogenate was then centrifuged at 18,000*g* for 10 min to remove cell debris. The resulting supernatant was collected for the assay. To account for background absorbance from glucose present in the samples, control reactions without the hydrolysis enzyme mixture were conducted for each sample. This ensured accurate determination of glycogen content.

### Liver mitochondria isolation and Seahorse analysis

Mouse liver mitochondria were isolated using an approach based on differential centrifugation. Briefly, half of the left lateral lobe was minced and homogenized using gentleMACS C Tubes (130-093-237, Miltenyi) on the gentleMACS Octo Dissociator (130-095-937, Miltenyi) with the m_mito_tissue_01 program in mitochondrial isolation buffer [70 mM sucrose, 210 mM mannitol, 5 mM Hepes, 1 mM EGTA, and 0.5% (w/v) fatty acid–free BSA (pH 7.2)]. The resultant homogenates were centrifuged twice at 1000*g* for 10 min at 4°C, and the supernatant was collected, which was centrifuged once at 8000*g* and 4°C for 10 min for mitochondrial enrichment. The pellets were resuspended in mitochondrial isolation buffer, and total protein was quantified by Pierce BCA Protein Assay Kit (23225, Thermo Fisher Scientific). Isolated mitochondria were resuspended in mitochondrial assay solution [70 mM sucrose, 220 mM mannitol, 10 mM KH_2_PO_4_, 5 mM MgCl_2_, 2 mM Hepes, 1 mM EGTA, and 0.2% (w/v) fatty acid–free BSA (pH 7.2)] to a final protein concentration of 0.2 mg/ml, and 50 μl was plated to each well (with background correction wells being an exception). The plate was centrifuged at 2000*g* for 20 min at 4°C, and 450 μl (or 500 μl for background wells) of mitochondrial assay solution plus substrates (pH 7.2, 37°C) was added to each well. To block CI and stimulate CII, 2 μM rotenone (R8875, Sigma-Aldrich) and 10 mM succinate (S9512, Sigma-Aldrich) were used as substrates, while 5 mM malate (M1000, Sigma-Aldrich) and 10 mM pyruvate (P2256, Sigma-Aldrich) were the substrates for CI activation. The XF24 sensor cartridge, previously hydrated with 500 μl of calibration buffer overnight at 37°C, was loaded with 40 mM adenosin diphosphate [ADP, from A5285 (pH 7.2)] at port A, oligomycin at port B (25 μg/ml), 40 μM carbonyl cyanide *p*-trfluoromethoxyphenylhydrazone (FCCP) at port C, and 40 μM of antimycin A/rotenone at port D (all from 103016-100, Agilent). The final concentrations in the wells were 4 mM ADP, oligomycin (2.5 μg/ml), 4 μM FCCP, and 4 μM antimycin A/rotenone. The plate was transferred to the XF24 instrument and OCR measured. Data were normalized for mitochondrial DNA of each well using the CyQUANT Cell Proliferation Assay Kit (C7026, Invitrogen) following the manufacturer’s instructions and analyzed using the Wave software. Three mice per genotype were used as biological replicates.

### Mitochondrial analysis through transmission electron microscopy

The liver was cut into pieces of about 1 mm^3^ and fixed with 2.5% glutaraldehyde and 2% paraformaldehyde in phosphate buffer (0.1 M, pH 7). The sample/fixation solution volume ratio was more than 1/10, and the fixation solution was replaced until it had a transparent color without evident blood. Hepatic samples were maintained in the same fixative solution for 24 more hours at 4°C. They were then washed with phosphate buffer (0.1 M, pH 7) and postfixed with 1% osmium tetroxide in the same buffer containing 0.8% potassium ferricyanide at 4°C. Next, the samples were dehydrated in acetone, infiltrated with Epon resin (Electron Microscopy Sciences) for 2 days, embedded in the same resin, orientated for longitudinal sectioning, and polymerized at 60°C for 48 hours. Semithin sections were performed under the light microscope to corroborate that the orientation was satisfactory. Then, the Leica Ultracut UC7 ultramicrotome (Leica Microsystems, Vienna) was used to obtain ultrathin sections, which were mounted on Formvar-coated copper grids. They were stained with UA-Zero (Agar) and lead citrate and then observed under a JEM-1010 electron microscope (Jeol) equipped with a charge-coupled device camera SIS Megaview III and the AnalySIS software at the Scientific and Technological Centers (CCiTUB), Universitat de Barcelona. Images were analyzed using the ImageJ software by manually identifying the mitochondria. Between 21 and 29 images were analyzed per mouse (four per genotype), totaling 215 images and 3885 mitochondria. The parameters under study were area, perimeter, circularity, and number of mitochondria per image.

### Isolation and culture of murine primary hepatocytes

Murine primary hepatocytes were isolated following the protocol described by Poggel *et al.* (*54*) with the Liver Perfusion kit (130-128-030, Miltenyi Biotec) in the gentleMACS Octo Dissociator with Heaters (130-096-427, Miltenyi Biotec). Briefly, the left lateral lobe of the liver was rapidly isolated from the mice and maintained in MACS Tissue Storage Solution (130-100-008, Miltenyi Biotec) supplemented with reagent E (1:200) on ice if not processed at the moment. The liver lobe was washed with sterile PBS and subjected to washes with predigestion buffer in the gentleMACS Perfuser (130-128-151, Miltenyi Biotec) attached to the gentleMACS Perfusion Sleeves (130-128-752, Miltenyi Biotec). Subsequently, after equilibration, enzymatic perfusion and digestion were performed, followed by filtration on a 100-μm strainer and a density gradient centrifugation using debris removal solution (130-109-398, Miltenyi Biotec). The pellet of hepatocytes was washed with attachment medium, a 1:1 mix of Dulbecco’s modified Eagle’s medium (DMEM) high glucose (SH30081.01, Hyclone) with HAM’S F12 (1176-054, Gibco) supplemented with 5 mM sodium pyruvate (11360-039, Gibco), 2 mM glutamine (GlutaMAX-I 100X, 35050-061, Gibco), 0.05% NaHCO_3_ (6329, Merck), 20 mM Hepes (SH40003.01, Cytiva), 1% antibiotic/antimicotic solution (SV30079.01, Cytiva), 20.6 mM (final concentration) glucose (158968, Sigma-Aldrich), 10% fetal bovine serum (FBS) (SH30071.03, Cytiva), 0.02% BSA (A7030-100, Sigma-Aldrich), and 0.1% plasmocyn (ant-mpt, InvivoGen). Cells were then seeded in the same medium in collagen-coated six-well plates at a density of 5*10^5^ cells per well and maintained in the same medium overnight. For RNA-seq, mRNA, and protein analysis, the cells were directly harvested. For the gluconeogenesis experiment, attachment medium was replaced by DMEM without glucose, glutamine, and sodium pyruvate (A1443001, Gibco) plus 5.5 mM glucose and 0.2% BSA for 3 hours. Then, the medium was changed to DMEM supplemented with BSA 0.2% and gluconeogenic precursors: 20 mM sodium pyruvate, 20 mM sodium lactate (71720, Sigma-Aldrich), and 2 mM glutamax. Conditioned medium and cells were harvested after 4 hours for further analyses. Conditioned medium was centrifuged at 400*g* for 5 min to remove cell remnants and tested for glucose concentration using the Glucose Assay Kit (ab65333, Abcam), and cells were used for protein extraction, quantification, and normalization of results.

### AML12 and THLE-2 cell culture

AML12 cells, which are hepatocytes isolated from the normal liver of a 3-month-old mouse, were obtained from the American Type Culture Collection (CRL-2254, ATCC) and were cultured in a humidified 5% CO_2_ incubator at 37°C with DMEM/F12 medium (31330-038, Gibco) supplemented with 10% FBS (SH30071.03, Cytiva), 1% antibiotic/antimycotic solution (SV30079.01, Cytiva), dexamethasone (40 ng/ml; D2915-100MG, Sigma-Aldrich), 1% insulin-transferrin-sodium selenite media (3146-5ML, Sigma-Aldrich) and 0.1% plasmocyn (ant-mpt, InvivoGen).

Human THLE-2 cells (ATCC CRL-2706) were purchased from ATCC and cultured in Bronchial Epithelial Basal Medium (BEBM) (CC-3171, Lonza) supplemented with bovine pituitary extract (BPE) (CC-4009F, Lonza), insulin (CC-4021F, Lonza), hydrocortisone (CC-4031F, Lonza), retinoic acid (CC-4085F, Lonza), transferrin (CC-4205F, Lonza), triiodothyronine (CC-4211F, Lonza), hEGF (CC-4230F, Lonza), epidermal growth factor (6 ng/ml; 354052, Corning), phosphoethanolamine (80 ng/ml; P-0503-1G, Sigma-Aldrich), 1% antibiotic/antimycotic solution (SV30079.01, Cytiva), and 10% FBS (SH30071.03, Cytiva) in a humidified 5% CO_2_ incubator at 37°C in precoated flasks. Coating was performed with plain BEBM medium supplemented with BSA (0.01 mg/ml; A7030-100, Sigma-Aldrich), fibronectin (0.01 mg/ml; F-2006, Sigma-Aldrich), and type I bovine collagen (0.03 mg/ml; 804592, Sigma-Aldrich) in a humidified 5% CO_2_ incubator at 37°C.

### Experiments with glucose, 2-deoxy-d-glucose, and glutamine

AML12 cells were seeded into 12-well plates at 200,000 cells per well and maintained in the supplemented DMEM-F12 medium mentioned above overnight (o/n). Medium was then changed to DMEM-F12 (31330-038, Gibco), only supplemented with 10% FBS (SH30071.03, Cytiva) and 1% antibiotic/antimycotic solution (SV30079.01, Cytiva) for 24 hour. Next, the cells were deprived for 1 hour in DMEM (A14430-01, Gibco) supplemented with 0.2% BSA (A7030, Sigma-Aldrich) and 1% antibiotic/antimycotic solution (SV30079.01, Cytiva). Depending on the specific experiment, the cells were then changed to the same medium containing 0, 5.5, or 25 mM glucose (158968, Sigma-Aldrich), containing or not 10 mM 2-deoxy-d-glucose (D6134, Sigma-Aldrich), and containing or not 2 mM glutamine (GlutaMAX-I 100X, 35050-061, Gibco). After 24 hours of treatment, the conditioned medium was recovered, centrifuged at 400*g* for 5 min to remove cell remnants, and tested for succinate concentration using the EnzyChrom Succinate Assay Kit (ESNT-100, BioAssay Systems). The cells were used to study gene expression.

### Seahorse analysis in AML12, THLE2, and murine primary hepatocytes

AML12, THLE2, and murine primary hepatocytes isolated from WT, *Sucnr1^−/−^*, *Sucnr1*^fl/fl^, and Hep-*Sucnr1* KO mice were seeded the day before the experiment into 24-well Seahorse assay plates at 10,000 to 20,000 cells per well. The next day, the cells were treated before the mitochondrial respiration analysis. AML12 cells were treated with 1 μM *Sucnr1* siRNA (E-040762-01-0050, Dharmacon) or scramble (D-001910-10-50, Dharmacon) diluted in ACCELL siRNA delivery medium (B-005000, Dharmacon) for 24 hours. THLE-2 cells were treated with 1 μM SUCNR1 antagonist NF-56-EJ40 (*55*) (MedChemExpress) for 5 hours or with the same treatment but with the addition of 100 μM SUCNR1 agonist *cis*-epoxysuccinic acid (*56*) (E0449, Tokyo Chemical Industry) for the last 3 hours of treatment in the BEBM medium without added supplements or FBS. Murine primary hepatocytes were seeded in attachment medium overnight and not treated before Seahorse analysis. After the respective protocols, the hepatocytes were washed and incubated in assay medium [10 mM glucose, 2 mM l-glutamine, and 1 mM sodium pyruvate in Seahorse XF base medium (at pH 7.4), 103575-100, Agilent] for 45 to 60 min at 37°C (without CO_2_). For mitochondrial stress tests, the OCR and the ECAR were measured in a Seahorse XFe24 analyzer after the following injections: 1 μM oligomycin, 2 μM FCCP, and 0.5 μM rotenone/antimycin A (103015-100, Agilent). Data were normalized for cellular DNA of each well using the CyQUANT Cell Proliferation Assay Kit (C7026, Invitrogen) following the manufacturer’s instructions and analyzed using the Wave software. Three different passages or at least three mice per genotype were used as biological replicates.

### Mitochondrial membrane polarization

AML12 with silenced *Sucnr1* (via siRNA as performed above) and scrambled controls were used to assess the mitochondrial membrane potential, following the manufacturer’s directions (MAK160, Sigma-Aldrich). In brief, 10^6^ cells were seeded in six-well plates and silenced for 24 hours as previously described. Thereafter, the cells were detached and incubated with JC-10 dye loading solution for 45 min, spun at 1000 rpm for 4 min at room temperature, and resuspended in Pre-Sort Buffer (563503, BD FACS). The fluorescent signal generated by the JC-10 dye (MAK160A, Sigma-Aldrich) was detected via flow cytometry using logarithmic amplification of 633/635-nm excitation and a red emission filter. A total of 50,000 events were counted using a low flow rate during acquisition.

### Isotope-based flux analysis

Murine primary hepatocytes from *Sucnr1*^fl/fl^ and Hep-*Sucnr1* KO mice were seeded in six-well collagen-coated plates overnight in attachment medium. For both experiments, the medium was changed to plain DMEM without glucose, without glutamine, and without sodium pyruvate (A1443001, Gibco) for 1 hour. For the ^13^C-glucose experiment, 5.5 mM ^13^C-glucose (389374, Sigma-Aldrich) was then added for 30 min. For the ^13^C-glutamine experiment, the medium was changed to plain DMEM (A1443001, Gibco) supplemented with 5.5 mM glucose and 2 mM ^13^C-glutamine (HY-N0390S1, MedChemExpress) for 1 hour. The cells were then washed in PBS. Metabolites were extracted from cells by adding 300 μl of cold methanol:water (8:1, v/v) containing d_3_-leucine as an internal standard (5 parts per million final concentration). The samples were vortexed and subjected to three freeze-thaw cycles using liquid nitrogen and then stored at −20°C for 1 hour. After centrifugation at 12,000 rpm for 10 min at 4°C, the supernatants were transferred to glass vials for LC-MS analysis.

Metabolomic profiling was performed on a Thermo Fisher Scientific Orbitrap IDX Tribrid mass spectrometer equipped with a heated electrospray ionization interface and coupled to a Vanquish UHPLC system. Chromatographic separation was achieved using a Waters ACQUITY Premier BEH Amide column (2.1 mm by 100 mm, 1.7 μm). The mobile phases were acetonitrile (A) and 5 mM ammonium acetate in water (B). The gradient program started at 10% B (1 min isocratic), increased to 50% B over 4.5 min, and then to 70% B, followed by reequilibration to 10% B within 0.5 min and held for 3 min; total run time was 10 min.

Column temperature was maintained at 25°C, with a flow rate of 0.4 ml/min and an injection volume of 8 μl. MS was performed in negative ion mode (full scan) using the following parameters: mass/charge ratio (*m*/*z*) range, 70 to 300; spray voltage, −2.8 kV; ion transfer tube and vaporizer temperatures both at 300°C; sheath gas, auxiliary gas, and sweep gas flow rates at 40, 10, and 1 a.u., respectively; S-lens RF level, 60%; resolution, 60,000 (at *m*/*z* 200); AGC target, 2 × 10^5^; and maximum injection time set to Auto. Instrument control and data acquisition were conducted using Xcalibur 4.4 software.

The monitored metabolites (*m*/*z*, retention time, and number of isotopologs) as well as the internal standard are listed in table S4. Total abundance was normalized to the amount of protein in each sample to obtain normalized abundance, and fractional contribution was calculated as the percentage of labeled C in a molecule.

### Gene expression analysis through reverse transcription qPCR

Tissue and cell samples were homogenized and lysed using TRIzol Reagent (15596018, Invitrogen), followed by chloroform extraction. Isopropanol was used to precipitate the RNA, which was then washed with ethanol and dissolved in ribonuclease (RNase)–free water. RNA was quantified with a μDrop Plate (N12391, Thermo Fisher Scientific) on a Varioskan Lux instrument, using the Skanit Microplate Reader software (Thermo Fisher Scientific). The 260:280 ratio was used to assess RNA purity. RNA was then retrotranscribed into cDNA using the High-Capacity cDNA Reverse Transcription Kit with a mix of random primers, deoxyribonucleoside triphosphates, RNase inhibitor, nuclease-free water, and MultiScribe Reverse Transcriptase (all from Applied Biosystems). The expression of target and housekeeping genes was evaluated for each sample by quantitative polymerase chain reaction (qPCR) using the TaqMan Fast Advanced Master Mix (4444554, Thermo Fisher Scientific) and the specific TaqMan probes (table S5) on a Quant Studio 7 Pro instrument (from Applied Biosystems). Results were normalized to *B2m* using the comparative CT method (2^-ΔΔCT^).

### Hepatocyte RNA-seq

Murine primary hepatocytes from *Sucnr1*^fl/fl^ and Hep-*Sucnr1* KO mice were seeded in six-well collagen-coated plates overnight in attachment media. Cells were then lysed, and RNA was extracted and purified with the RNeasy Mini Kit (74104, Qiagen). Total RNA extractions were quantified using a Nanodrop One (Thermo Fisher Scientific), and RNA integrity was assessed with the Bioanalyzer 2100 RNA Nano assay (Agilent). Libraries for RNA-seq were prepared at IRB Barcelona Functional Genomics Core Facility. Briefly, mRNA was isolated from 1.1 μg of total RNA and used to generate dual-indexed cDNA libraries with the Illumina stranded mRNA ligation kit (Illumina) and UD Indexes Set A (Illumina). Ten cycles of PCR amplification were applied to all libraries.

Sequencing-ready libraries were quantified using the Qubit dsDNA HS assay (Invitrogen) and quality controlled with the Bioanalyzer 2100 DNA HS assay (Agilent). An equimolar pool was prepared with the six libraries and submitted for sequencing on a NovaSeq6000 S4 (Illumina). More than 71 Gbp were produced, with a minimum of 36.5 million paired-end reads per sample.

Stranded paired-end reads were processed using Trimgalore (v0.6.7) (*57*) using default parameters. Trimmed reads were aligned to the mouse reference genome version GRCm38 using STAR (v2.7.10a) (*58*) with default parameters. STAR indexes were built using the ENSEMBL annotation version GRCm38.102. SAM files were converted to BAM and sorted using sambamba (v2.098.1) (*59*). Gene counts were obtained with the featureCounts function from the Rsubread package (*60*) with the gtf file corresponding to ENSEMBL version GRCm38.102 and parameters set to isPairedEnd = TRUE and strandSpecific = 2. All RNA-seq data have been uploaded to Gene Expression Omnibus: GSE301098. Analyses were performed in the R programming language version 4.1.3 (*61*) unless otherwise stated. Differential analyses were performed using the DESeq2 package (*62*) with the experimental date of extraction as covariate.

Pathway analysis was conducted on the gene log_2_ fold changes via the GSEA method with the following parameters: clusterProfiler (version 2.1.6) (*52*) and PathwayPA (version 1.48.0) (*53*) for R libraries, default parameters of minimal gene set (column setSize) size to be included for analysis (pericentral, periportal, and Wnt), significance filter of *q* value <0.05, ranked by *q* values, and, in case of exact *q* value, ranked by absolute NES value. Pericentral, periportal, and Wnt gene sets were extracted from Plata-Gómez *et al.* (*15*) (table S2), from which the baseMean ≤5 filter was applied to remove minimally expressed genes.

GO overrepresentation analysis was performed on periportal, pericentral, and Wnt gene sets, using the clusterProfiler R package (version 4.12.6). Significantly enriched GO terms were then semantically clustered into parent GO terms, using the rrvgo R package (version 1.16.0). The relationship between enriched parent GO terms and the periportal, pericentral, and Wnt gene sets was represented with chord diagrams, using the circlize R package (version 0.4.16). Connection widths are proportional to the absolute log_2_ fold change of each gene. For visual clarity, only the top five up-regulated or down-regulated genes per parent GO term are represented. Analysis parameters are as follows: org.Mm.eg.db annotation database (version 3.19.1), minimum gene set size of 5, *P* value cutoff of 0.01, semantic similarity threshold of 0.8, and similarity method “relative.”

### Western blot

Liver tissue (20 mg) or cells (5 × 10^5^) were lysed in Mammalian Protein Extraction Reagent (M-PER, 78501) supplemented with Halt Protease (1861278) and Phosphatase Inhibitor Cocktail (1861277) plus EDTA (1861275) (all from Thermo Fisher Scientific). Tissue and cell lysates were then centrifuged at 13,000 rpm for 10 min at 4°C to remove cell debris, and protein was quantified using the Pierce BCA Protein Assay Kit (23225, Thermo Fisher Scientific). For each sample, 15 (cells) 20 to 50 (tissue) μg of protein were loaded onto 8% or 10% SDS polyacrylamide gels for electrophoresis, followed by transfer to polyvinylidene difluoride membranes. Five % (w/v) nonfat dry milk or 5% (w/v) BSA in 1× tris-buffered saline 0.1% Tween 20 was used to block membranes, which were then incubated overnight at 4°C with the specific primary antibodies (refer to table S6). Following washes, the membranes were incubated for 1 hour at room temperature with the appropriate secondary antibody at a 1:2000 dilution (table S6). The Immobilon ECL Ultra Western HRP Substrate (WBULS0500, EMD Millipore Corporation) and the iBright 1500 system (Thermo Fisher Scientific) were used to visualize protein bands, which were analyzed using ImageJ software (NIH). Band intensities were normalized to loading controls (β-actin or glyceraldehyde-3-phosphate dehydrogenase).

### Statistics

In all the experiments, normality was assessed with the Shapiro-Wilk test. In the two-group comparisons, significant differences between normal distributions were determined using unpaired *t* tests (two-tailed, 95% confidence interval), and the Mann-Whitney test was used for nonnormal distributions. One-way analysis of variance (ANOVA) plus Tukey’s or Šídák’s multiple comparisons test was used to analyze the differences between more than two groups with normal distribution, and Kruskal-Wallis plus Dunn’s multiple comparison test was used for more than two groups with a nonnormal distribution. Two-way ANOVA was used to test statistical significance in the GTT, PTT, ITT, and the fasting experiment. The ROUT method (*Q* = 1%) from GraphPad Prism was used to identify outliers. Scatter dot plots and time-course graphs data are presented in graphs as means ± SEM. The results were considered significant at *P* < 0.05. GraphPad Prism 9 software was used for statistical analysis and graphical representations. See Liver proteomics and Hepatocytes RNA-seq for specific statistical analyses and graph representation.

## Notes

**Correction (16 November 2026):** In the original version of the article, a paper that describes the generation of the *Sucnr1*^−/−^ mouse model used in the study was inadvertently omitted from the reference list. The following reference has been added as ref. (*49*) and cited in the section “Global *Sucnr1* KO (*Sucnr1*^−/−^) mice”: K. J. McCreath, S. Espada, B. G. Gálvez, M. Benito, A. de Molina, P. Sepúlveda, A. M. Cervera, Targeted disruption of the SUCNR1 metabolic receptor leads to dichotomous effects on obesity. *Diabetes* **64**, 1154–1167 (2015). Subsequent references were renumbered. The online and PDF versions of the article have been updated.

## References

[1] T. Martini, F. Naef, J. S. Tchorz, Spatiotemporal metabolic liver zonation and consequences on pathophysiology. Annu. Rev. Pathol. 18, 439–466 (2023). 10.1146/annurev-pathmechdis-031521-024831 36693201

[2] S. W. S. Kang, R. P. Cunningham, C. B. Miller, L. A. Brown, C. M. Cultraro, A. Harned, K. Narayan, J. Hernandez, L. M. Jenkins, A. Lobanov, M. Cam, N. Porat-Shliom, A spatial map of hepatic mitochondria uncovers functional heterogeneity shaped by nutrient-sensing signaling. Nat. Commun. 15, 1799 (2024). 10.1038/s41467-024-45751-9 PMC10902380 38418824

[3] S. Fernández-Veledo, A. Marsal-Beltran, J. Vendrell, Type 2 diabetes and succinate: Unmasking an age-old molecule. Springer Science and Business Media Deutschland GmbH [Preprint] (2024); 10.1007/s00125-023-06063-7. PMC10844351 38182909

[4] S. Fernández-Veledo, V. Ceperuelo-Mallafré, J. Vendrell, Rethinking succinate: An unexpected hormone-like metabolite in energy homeostasis. Trends Endocrinol. Metab. 32, 680–692 (2021). 10.1016/j.tem.2021.06.003 34301438

[5] S. Fernández-Veledo, J. Vendrell, Gut microbiota-derived succinate: Friend or foe in human metabolic diseases? Rev. Endocr. Metab. Disord. 20, 439–447 (2019). 10.1007/s11154-019-09513-z PMC6938788 31654259

[6] C. Serena, V. Ceperuelo-Mallafré, N. Keiran, M. I. Queipo-Ortuño, R. Bernal, R. Gomez-Huelgas, M. Urpi-Sarda, M. Sabater, V. Pérez-Brocal, C. Andrés-Lacueva, A. Moya, F. J. Tinahones, J. M. Fernández-Real, J. Vendrell, S. Fernández-Veledo, Elevated circulating levels of succinate in human obesity are linked to specific gut microbiota. ISME J. 12, 1642–1657 (2018). 10.1038/s41396-018-0068-2 PMC6018807 29434314

[7] V. Ceperuelo-Mallafre, G. Llaurado, N. Keiran, E. Benaiges, B. Astiarraga, L. Martinez, S. Pellitero, J. M. Gonzalez-Clemente, A. Rodriguez, J. M. Fernandez-Real, A. Lecube, A. Megia, N. Vilarrasa, J. Vendrell, S. Fernandez-Veledo, Preoperative circulating succinate levels as a biomarker for diabetes remission after bariatric surgery. Diabetes Care 42, 1956–1965 (2019). 10.2337/dc19-0114 31375523

[8] J. A. van Diepen, J. H. Robben, G. J. Hooiveld, C. Carmone, M. Alsady, L. Boutens, M. Bekkenkamp-Grovenstein, A. Hijmans, U. F. H. Engelke, R. A. Wevers, M. G. Netea, C. J. Tack, R. Stienstra, P. M. T. Deen, SUCNR1-mediated chemotaxis of macrophages aggravates obesity-induced inflammation and diabetes. Diabetologia 60, 1304–1313 (2017). 10.1007/s00125-017-4261-z PMC5487589 28382382

[9] A. Marsal-Beltran, A. Rodríguez-Castellano, B. Astiarraga, E. Calvo, P. Rada, A. Madeira, M.-M. Rodríguez-Peña, G. Llauradó, C. Núñez-Roa, B. Gómez-Santos, E. Maymó-Masip, R. Bosch, M. D. Frutos, J. M. Moreno-Navarrete, B. Ramos-Molina, P. Aspichueta, J. Joven, J.-M. Fernández-Real, J. C. Quer, Á. M. Valverde, A. Pardo, J. Vendrell, V. Ceperuelo-Mallafré, S. Fernández-Veledo, Protective effects of the succinate/SUCNR1 axis on damaged hepatocytes in NAFLD. Metabolism 145, 155630 (2023). 10.1016/j.metabol.2023.155630 37315889

[10] S. Chashmniam, M. Ghafourpour, A. Rezaei Farimani, A. Gholami, B. F. Nobakht Motlagh Ghoochani, Metabolomic biomarkers in the diagnosis of non-alcoholic fatty liver disease. Hepat. Mon. 19, e92244 (2019). PMC6536017 31191836

[11] R. Loomba, V. Seguritan, W. Li, T. Long, N. Klitgord, A. Bhatt, P. S. Dulai, C. Caussy, R. Bettencourt, S. K. Highlander, M. B. Jones, C. B. Sirlin, B. Schnabl, L. Brinkac, N. Schork, C.-H. Chen, D. A. Brenner, W. Biggs, S. Yooseph, J. C. Venter, Gut microbiome based metagenomic signature for non-invasive detection of advanced fibrosis in human nonalcoholic fatty liver disease. Cell Metab. 25, 1054–1062.e5 (2017). 10.1016/j.cmet.2017.04.001 PMC5502730 28467925

[12] B. Astiarraga, L. Martínez, V. Ceperuelo-Mallafré, G. Llauradó, M. Terrón-Puig, M. M. Rodríguez, A. Casajoana, S. Pellitero, A. Megía, N. Vilarrasa, J. Vendrell, S. Fernández-Veledo, Impaired succinate response to a mixed meal in obesity and type 2 diabetes is normalized after metabolic surgery. Diabetes Care 43, 2581–2587 (2020). 10.2337/dc20-0460 PMC7510048 32737141

[13] T. Villanueva-Carmona, L. Cedó, A. Madeira, V. Ceperuelo-Mallafré, M.-M. Rodríguez-Peña, C. Núñez-Roa, E. Maymó- Masip, M. Repollés-de-Dalmau, J. Badia, N. Keiran, M. Mirasierra, C. Pimenta-Lopes, J. Sabadell-Basallote, R. Bosch, L. Caubet, J. C. Escolà- Gil, J.-M. Fernández-Real, N. Vilarrasa, F. Ventura, M. Vallejo, J. Vendrell, S. Fernández-Veledo, SUCNR1 signaling in adipocytes controls energy metabolism by modulating circadian clock and leptin expression. Cell Metab. 35, 601–619.e10 (2023). 10.1016/j.cmet.2023.03.004 36977414

[14] J. Sabadell-Basallote, B. Astiarraga, C. Castaño, M. Ejarque, M. Repollés-De-Dalmau, I. Quesada, J. Blanco, C. Núñez-Roa, M. M. Rodríguez-Peña, L. Martínez, D. F. De Jesus, L. Marroquí, R. Bosch, E. Montanya, F. X. Sureda, A. Tura, A. Mari, R. N. Kulkarni, J. Vendrell, S. Fernández-Veledo, SUCNR1 regulates insulin secretion and glucose elevates the succinate response in people with prediabetes. J. Clin. Invest. 134, e173214 (2024). 10.1172/JCI173214 PMC11178533 38713514

[15] A. B. Plata-Gómez, L. de Prado-Rivas, A. Sanz, N. Deleyto-Seldas, F. García, C. de la Calle Arregui, C. Silva, E. Caleiras, O. Graña-Castro, E. Piñeiro-Yáñez, J. Krebs, L. Leiva-Vega, J. Muñoz, A. Jain, G. Sabio, A. Efeyan, Hepatic nutrient and hormone signaling to mTORC1 instructs the postnatal metabolic zonation of the liver. Nat. Commun. 15, 1878 (2024). 10.1038/s41467-024-46032-1 PMC10948770 38499523

[16] S. Wang, B. Xu, J. Liang, Y. Feng, P. Han, J. Shen, X. Li, M. Zheng, T. Zhang, C. Zhang, P. Mi, Y. Zhang, Z. Liu, S. Li, D. Yuan, Spatial transcriptomic study reveals heterogeneous metabolic adaptation and a role of pericentral PPARα/CAR/Ces2a axis during fasting in mouse liver. Adv. Sci. 11, e2405240 (2024). 10.1002/advs.202405240 PMC11538668 39234807

[17] N. Keiran, V. Ceperuelo-Mallafré, E. Calvo, M. I. Hernández-Alvarez, M. Ejarque, C. Núñez-Roa, D. Horrillo, E. Maymó-Masip, M. M. Rodríguez, R. Fradera, J. V. de la Rosa, R. Jorba, A. Megia, A. Zorzano, G. Medina-Gómez, C. Serena, A. Castrillo, J. Vendrell, S. Fernández-Veledo, SUCNR1 controls an anti-inflammatory program in macrophages to regulate the metabolic response to obesity. Nat. Immunol. 20, 581–592 (2019). 10.1038/s41590-019-0372-7 30962591

[18] E. Gaude, C. Schmidt, P. A. Gammage, A. Dugourd, T. Blacker, S. P. Chew, J. Saez-Rodriguez, J. S. O’Neill, G. Szabadkai, M. Minczuk, C. Frezza, NADH shuttling couples cytosolic reductive carboxylation of glutamine with glycolysis in cells with mitochondrial dysfunction. Mol. Cell 69, 581–593.e7 (2018). 10.1016/j.molcel.2018.01.034 PMC5823973 29452638

[19] M. P. Murphy, E. T. Chouchani, Why succinate? Physiological regulation by a mitochondrial coenzyme Q sentinel. Nat. Chem. Biol. 18, 461–469 (2022). 10.1038/s41589-022-01004-8 PMC9150600 35484255

[20] D. Inverso, J. Shi, K. H. Lee, M. Jakab, S. Ben-Moshe, S. R. Kulkarni, M. Schneider, G. Wang, M. Komeili, P. A. Vélez, M. Riedel, C. Spegg, T. Ruppert, C. Schaeffer-Reiss, D. Helm, I. Singh, M. Boutros, S. Chintharlapalli, M. Heikenwalder, S. Itzkovitz, H. G. Augustin, A spatial vascular transcriptomic, proteomic, and phosphoproteomic atlas unveils an angiocrine Tie–Wnt signaling axis in the liver. Dev. Cell 56, 1677–1693.e10 (2021). 10.1016/j.devcel.2021.05.001 PMC8191494 34038707

[21] K. B. Halpern, R. Shenhav, H. Massalha, B. Toth, A. Egozi, E. E. Massasa, C. Medgalia, E. David, A. Giladi, A. E. Moor, Z. Porat, I. Amit, S. Itzkovitz, Paired-cell sequencing enables spatial gene expression mapping of liver endothelial cells. Nat. Biotechnol. 36, 962–970 (2018). 10.1038/nbt.4231 PMC6546596 30222169

[22] Y. Liang, K. Kaneko, B. Xin, J. Lee, X. Sun, K. Zhang, G. S. Feng, Temporal analyses of postnatal liver development and maturation by single-cell transcriptomics. Dev. Cell 57, 398–414.e5 (2022). 10.1016/j.devcel.2022.01.004 PMC8842999 35134346

[23] A. Reddy, L. H. M. Bozi, O. K. Yaghi, E. L. Mills, H. Xiao, H. E. Nicholson, M. Paschini, J. A. Paulo, R. Garrity, D. Laznik-Bogoslavski, J. C. B. Ferreira, C. S. Carl, K. A. Sjøberg, J. F. P. Wojtaszewski, J. F. Jeppesen, B. Kiens, S. P. Gygi, E. A. Richter, D. Mathis, E. T. Chouchani, pH-gated succinate secretion regulates muscle remodeling in response to exercise. Cell 183, 62–75.e17 (2020). 10.1016/j.cell.2020.08.039 PMC7778787 32946811

[24] Q. Chen, K. Kirk, Y. I. Shurubor, D. Zhao, A. J. Arreguin, I. Shahi, F. Valsecchi, G. Primiano, E. L. Calder, V. Carelli, T. T. Denton, M. F. Beal, S. S. Gross, G. Manfredi, M. D’Aurelio, Rewiring of glutamine metabolism is a bioenergetic adaptation of human cells with mitochondrial DNA mutations. Cell Metab. 27, 1007–1025.e5 (2018). 10.1016/j.cmet.2018.03.002 PMC5932217 29657030

[25] R. J. Deberardinis, A. Mancuso, E. Daikhin, I. Nissim, M. Yudkoff, S. Wehrli, C. B. Thompson, Beyond aerobic glycolysis: Transformed cells can engage in glutamine metabolism that exceeds the requirement for protein and nucleotide synthesis. Proc. Natl. Acad. Sci. U.S.A. 104, 19345–19350 (2007). 10.1073/pnas.0709747104 PMC2148292 18032601

[26] M. G. V. Heiden, L. C. Cantley, C. B. Thompson, Understanding the Warburg effect: The metabolic requirements of cell proliferation. Science 324, 1029–1033 (2009). 10.1126/science.1160809 PMC2849637 19460998

[27] S. R. Nagarajan, M. Paul-Heng, J. R. Krycer, D. J. Fazakerley, A. F. Sharland, X. Andrew, J. Hoy, Lipid and glucose metabolism in hepatocyte cell lines and primary mouse hepatocytes: A comprehensive resource for in vitro studies of hepatic metabolism. Am. J. Physiol. Endocrinol. Metab. 316, 578–589 (2019). 10.1152/ajpendo.00365.2018 30694691

[28] R. Uzhachenko, K. Boyd, D. Olivares-Villagomez, Y. Zhu, J. S. Goodwin, T. Rana, A. Shanker, W. J. T. Tan, T. Bondar, R. Medzhitov, A. V. Ivanova, Mitochondrial protein Fus1/Tusc2 in premature aging and age-related pathologies: Critical roles of calcium and energy homeostasis. Aging 9, 627–649 (2017). 10.18632/aging.101213 PMC5391223 28351997

[29] A. S. Divakaruni, M. Jastroch, A practical guide for the analysis, standardization and interpretation of oxygen consumption measurements. Nat. Metab. 4, 978–994 (2022). 10.1038/s42255-022-00619-4 PMC9618452 35971004

[30] M. Liu, N. Ma, S. Li, Z. Kang, M. Wang, D. Wang, J. Zhao, H. Jiao, Y. Zhou, X. Wang, H. Li, H. Lin, Prevotella-produced succinate alleviates hepatic steatosis by enhancing mitochondrial function in layer-type chickens. J. Nutr. 155, 1751–1767 (2025). 10.1016/j.tjnut.2025.04.018 40274237

[31] A. D. Liebing, P. Rabe, P. Krumbholz, C. Zieschang, F. Bischof, A. Schulz, S. Billig, C. Birkemeyer, T. Pillaiyar, M. Garcia-Marcos, R. Kraft, C. Stäubert, Succinate receptor 1 signaling mutually depends on subcellular localization and cellular metabolism. FEBS J. 292, 2017–2050 (2025). 10.1111/febs.17407 PMC12001207 39838520

[32] Y. Zhang, H. Gong, L. Jin, P. Liu, J. Fan, X. Qin, Q. Zheng, Succinate predisposes mice to atrial fibrillation by impairing mitochondrial function via SUCNR1/AMPK axis. Redox Biol. 81, 103576 (2025). 10.1016/j.redox.2025.103576 PMC11915173 40031129

[33] P. Rabe, A. D. Liebing, P. Krumbholz, R. Kraft, C. Stäubert, Succinate receptor 1 inhibits mitochondrial respiration in cancer cells addicted to glutamine. Cancer Lett. 526, 91–102 (2022). 10.1016/j.canlet.2021.11.024 34813893

[34] M. Riou, A. L. Charles, I. Enache, C. Evrard, C. Pistea, M. Giannini, A. Charloux, B. Geny, Acute severe hypoxia decreases mitochondrial chain complex II respiration in human peripheral blood mononuclear cells. Int. J. Mol. Sci. 26, 705 (2025). 10.3390/ijms26020705 PMC11765662 39859418

[35] M. Grings, B. Seminotti, A. Karunanidhi, L. Ghaloul-Gonzalez, A.-W. Mohsen, P. Wipf, J. Palmfeldt, J. Vockley, G. Leipnitz, ETHE1 and MOCS1 deficiencies: Disruption of mitochondrial bioenergetics, dynamics, redox homeostasis and endoplasmic reticulum-mitochondria crosstalk in patient fibroblasts. Sci. Rep. 9, 12651 (2019). 10.1038/s41598-019-49014-2 PMC6718683 31477743

[36] L. Bramasole, A. Sinha, S. Gurevich, M. Radzinski, Y. Klein, N. Panat, E. Gefen, T. Rinaldi, D. Jimenez-Morales, J. Johnson, N. J. Krogan, N. Reis, D. Reichmann, M. H. Glickman, E. Pick, Proteasome lid bridges mitochondrial stress with Cdc53/Cullin1 NEDDylation status. Redox Biol. 20, 533–543 (2019). 10.1016/j.redox.2018.11.010 PMC6279957 30508698

[37] M. E. Fusakio, J. A. Willy, Y. Wang, E. T. Mirek, R. J. T. A. Baghdadi, C. M. Adams, T. G. Anthony, R. C. Wek, Transcription factor ATF4 directs basal and stress-induced gene expression in the unfolded protein response and cholesterol metabolism in the liver. Mol. Biol. Cell 27, 1536–1551 (2016). 10.1091/mbc.E16-01-0039 PMC4850040 26960794

[38] H. Kawecka, N. Imai, Y. Ohsaki, J. Cheng, D. Liu, J. Zhang, F. Mizuno, T. Tanaka, S. Yokoyama, K. Yamamoto, T. Ito, Y. Ishizu, T. Honda, T. Ishikawa, M. Woźniak, H. Wake, H. Kawashima, Morphological alterations of peridroplet mitochondria in human liver biopsy. Sci. Rep. 15, 38650 (2025). 10.1038/s41598-025-22496-z PMC12586439 41188377

[39] M. Morita, S. P. Gravel, V. Chénard, K. Sikström, L. Zheng, T. Alain, V. Gandin, D. Avizonis, M. Arguello, C. Zakaria, S. McLaughlan, Y. Nouet, A. Pause, M. Pollak, E. Gottlieb, O. Larsson, J. St-Pierre, I. Topisirovic, N. Sonenberg, MTORC1 controls mitochondrial activity and biogenesis through 4E-BP-dependent translational regulation. Cell Metab. 18, 698–711 (2013). 10.1016/j.cmet.2013.10.001 24206664

[40] K. Uehara, W. D. Lee, M. Stefkovich, D. Biswas, D. Santoleri, A. G. Whitlock, W. Quinn, T. Coopersmith, K. T. Creasy, D. J. Rader, K. Sakamoto, J. D. Rabinowitz, P. M. Titchenell, mTORC1 controls murine postprandial hepatic glycogen synthesis via *Ppp1r3b*. J. Clin. Investig. 134, e173782 (2024). 10.1172/JCI173782 PMC10977990 38290087

[41] E. G. Frame, The levels of individual free amino acids in the plasma of normal man at various intervals after a high-protein meal. J. Clin. Invest. 37, 1710–1723 (1958). 10.1172/JCI103763 PMC1062857 13611038

[42] L. M. Rogers, A. E. Belfield, M. Korzepa, A. Gritsas, T. A. Churchward-Venne, L. Breen, Postprandial plasma aminoacidemia and indices of appetite regulation following pea-rice blend, pea isolate and whey protein ingestion in healthy young adults. Br. J. Nutr. 132, 691–700 (2024). 10.1017/S0007114524001958 PMC11557286 39387207

[43] N. Ji, L. Xiang, B. Zhou, Y. Lu, M. Zhang, Hepatic gene expression profiles during fed–fasted–refed state in mice. Front. Genet. 14, 1145769 (2023). 10.3389/fgene.2023.1145769 PMC10020372 36936413

[44] C. H. Ang, P. Arandjelovic, J. Cheng, J. Yang, F. Guo, Y. Yu, S. Nelameham, L. Whitehead, J. Li, D. L. Silver, N. Barker, J. E. Visvader, P. K. H. Chow, G. K. Smyth, Y. Chen, D. M. Virshup, N. Y. Fu, Self-maintenance of zonal hepatocytes during adult homeostasis and their complex plasticity upon distinct liver injuries. Cell Rep. 44, 115093 (2025). 10.1016/j.celrep.2024.115093 39721024

[45] K. B. Halpern, R. Shenhav, O. Matcovitch-Natan, B. Tóth, D. Lemze, M. Golan, E. E. Massasa, S. Baydatch, S. Landen, A. E. Moor, A. Brandis, A. Giladi, A. Stokar-Avihail, E. David, I. Amit, S. Itzkovitz, Single-cell spatial reconstruction reveals global division of labour in the mammalian liver. Nature 542, 352–356 (2017). 10.1038/nature21065 PMC5321580 28166538

[46] H. Massalha, K. Bahar Halpern, S. Abu-Gazala, T. Jana, E. E. Massasa, A. E. Moor, L. Buchauer, M. Rozenberg, E. Pikarsky, I. Amit, G. Zamir, S. Itzkovitz, A single cell atlas of the human liver tumor microenvironment. Mol. Syst. Biol. 16, e9682 (2020). 10.15252/msb.20209682 PMC7746227 33332768

[47] S. Hu, S. Liu, Y. Bian, M. Poddar, S. Singh, C. Cao, J. McGaughey, A. Bell, L. L. Blazer, J. J. Adams, S. S. Sidhu, S. Angers, S. P. Monga, Single-cell spatial transcriptomics reveals a dynamic control of metabolic zonation and liver regeneration by endothelial cell Wnt2 and Wnt9b. Cell Rep. Med. 3, 100754 (2022). 10.1016/j.xcrm.2022.100754 PMC9588996 36220068

[48] A. Efeyan, R. Zoncu, S. Chang, I. Gumper, H. Snitkin, R. L. Wolfson, O. Kirak, D. D. Sabatini, D. M. Sabatini, Regulation of mTORC1 by the Rag GTPases is necessary for neonatal autophagy and survival. Nature 493, 679–683 (2013). 10.1038/nature11745 PMC4000705 23263183

[49] K. J. McCreath, S. Espada, B. G. Gálvez, M. Benito, A. de Molina, P. Sepúlveda, A. M. Cervera, Targeted disruption of the SUCNR1 metabolic receptor leads to dichotomous effects on obesity. Diabetes 64, 1154–1167 (2015). 10.2337/db14-0346 25352636

[50] M. E. Ritchie, B. Phipson, D. Wu, Y. Hu, C. W. Law, W. Shi, G. K. Smyth, *limma* powers differential expression analyses for RNA-sequencing and microarray studies. Nucleic Acids Res. 43, e47 (2015). 10.1093/nar/gkv007 PMC4402510 25605792

[51] Y. Majeed, N. Halabi, A. Y. Madani, R. Engelke, A. M. Bhagwat, H. Abdesselem, M. V. Agha, M. Vakayil, R. Courjaret, N. Goswami, H. Ben Hamidane, M. A. Elrayess, A. Rafii, J. Graumann, F. Schmidt, N. A. Mazloum, SIRT1 promotes lipid metabolism and mitochondrial biogenesis in adipocytes and coordinates adipogenesis by targeting key enzymatic pathways. Sci. Rep. 11, 8177 (2021). 10.1038/s41598-021-87759-x PMC8046990 33854178

[52] S. Xu, E. Hu, Y. Cai, Z. Xie, X. Luo, L. Zhan, W. Tang, Q. Wang, B. Liu, R. Wang, W. Xie, T. Wu, L. Xie, G. Yu, Using clusterProfiler to characterize multiomics data. Nat. Protoc. 19, 3292–3320 (2024). 10.1038/s41596-024-01020-z 39019974

[53] G. Yu, Q. Y. He, ReactomePA: An R/Bioconductor package for reactome pathway analysis and visualization. Mol. Biosyst. 12, 477–479 (2016). 10.1039/c5mb00663e 26661513

[54] C. Poggel, T. Adams, R. Janzen, A. Hofmann, O. Hardt, E. Roeb, S. K. Schröder, C. G. Tag, M. Roderfeld, R. Weiskirchen, Isolation of hepatocytes from liver tissue by a novel, semi-automated perfusion technology. Biomedicines 10, 2198 (2022). 10.3390/biomedicines10092198 PMC9496349 36140299

[55] M. Haffke, D. Fehlmann, G. Rummel, J. Boivineau, M. Duckely, N. Gommermann, S. Cotesta, F. Sirockin, F. Freuler, A. Littlewood-Evans, K. Kaupmann, V. P. Jaakola, Structural basis of species-selective antagonist binding to the succinate receptor. Nature 574, 581–585 (2019). 10.1038/s41586-019-1663-8 31645725

[56] P. Geubelle, J. Gilissen, S. Dilly, L. Poma, N. Dupuis, C. Laschet, D. Abboud, A. Inoue, F. Jouret, B. Pirotte, J. Hanson, Identification and pharmacological characterization of succinate receptor agonists. Br. J. Pharmacol. 174, 796–808 (2017). 10.1111/bph.13738 PMC5386996 28160606

[57] Felix Krueger, TrimGalore. https://github.com/FelixKrueger/TrimGalore.

[58] A. Dobin, C. A. Davis, F. Schlesinger, J. Drenkow, C. Zaleski, S. Jha, P. Batut, M. Chaisson, T. R. Gingeras, STAR: Ultrafast universal RNA-seq aligner. Bioinformatics 29, 15–21 (2013). 10.1093/bioinformatics/bts635 PMC3530905 23104886

[59] A. Tarasov, P. Prins, Sambamba v0.5.0. Zenodo (2014); 10.5281/zenodo.13200.

[60] Y. Liao, G. K. Smyth, W. Shi, The R package Rsubread is easier, faster, cheaper and better for alignment and quantification of RNA sequencing reads. Nucleic Acids Res. 47, e47 (2019). 10.1093/nar/gkz114 PMC6486549 30783653

[61] R Core Team, R: A language and environment for statistical computing. R Foundation for Statistical Computing, Vienna, Austria (2021); https://r-project.org/.

[62] M. I. Love, W. Huber, S. Anders, Moderated estimation of fold change and dispersion for RNA-seq data with DESeq2. Genome Biol. 15, 550 (2014). 10.1186/s13059-014-0550-8 PMC4302049 25516281

