# Supplementary material for: SUCNR1 coordinates metabolic flux, mitochondrial function, and nutrient-dependent adaptation in hepatocytes
Source: Sci Adv. 2026 Jun 12;12(24):eaec8873. doi: 10.1126/sciadv.aec8873 (PMC13262611; doi:10.1126/sciadv.aec8873)
Supplement: Supplementary file 1 — Figs. S1 to S10 Tables S1 to S6 Legends for data S1 to S15 [file sciadv.aec8873_sm.pdf]

Science Advances
AAAS

Supplementary Materials for

SUCNR1 coordinates metabolic flux, mitochondrial function, and nutrient-dependent adaptation in hepatocytes

Anna Marsal-Beltran *et al.*

Corresponding author: Victòria Ceperuelo-Mallafré, victoria.ceperuelo@urv.cat;
Sonia Fernández-Veledo, sonia.fernandez@irbcatsud.cat

Sci. Adv. **12**, eaec8873 (2026)
DOI: 10.1126/sciadv.aec8873

The PDF file includes:

Figs. S1 to S10
Tables S1 to S6
Legends for data S1 to S15

Other Supplementary Material for this manuscript includes the following:

Data S1 to S15

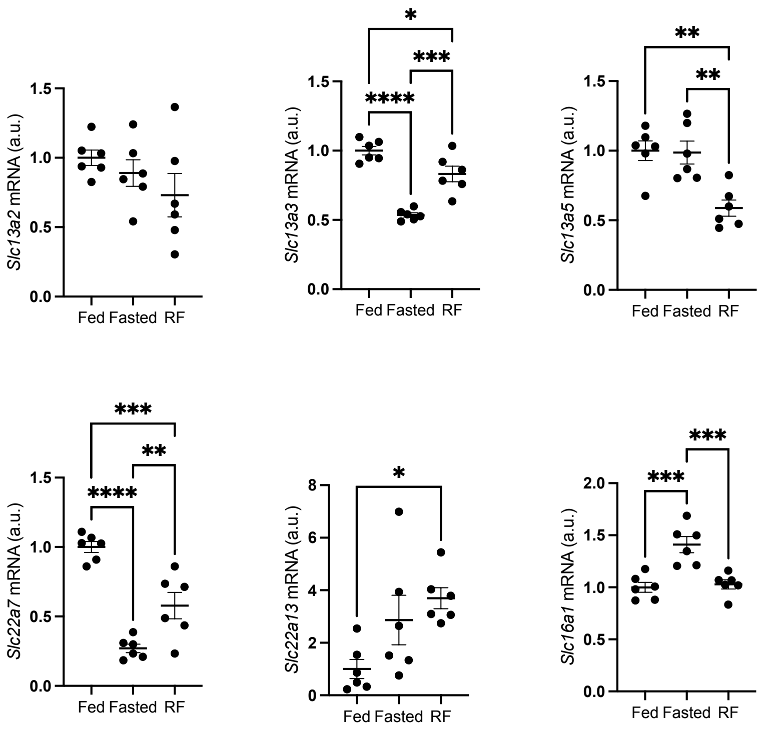

Fig. S1. Succinate transporters expression in response to fasting and refeeding. RT-qPCR of succinate transporters in livers of 8-12-week-old WT mice in three conditions: *ad libitum*-fed, fasted for 24h, or fasted for 24h +24h refed (RF) (n=5-6). Results are presented as mean ± SEM; * $p < 0.05$; ** $p < 0.01$; *** $p < 0.001$; **** $p < 0.0001$ (one-way ANOVA plus Tukey's multiple comparisons test).

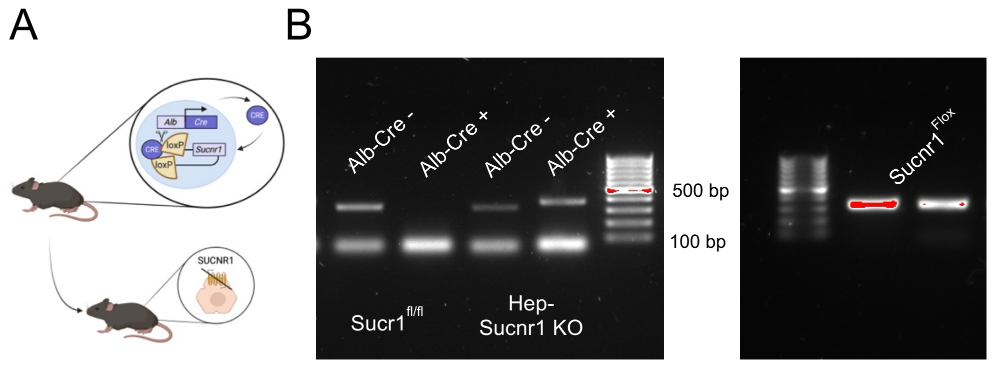

Fig. S2. Hep-*Sucnr1* KO mouse model generation and genotyping. Schematic representation of Cre recombinase action on loxP sequences in Hep-*Sucnr1* KO hepatocytes. Created in BioRender. Fernández-Veledo, S. (2026) https://BioRender.com/glod1lx (**A**). PCR products of the *Alb-Cre*$^{-}$, *Alb-Cre*$^{+}$ and *Sucnr1*$^{fl/fl}$ amplifications (**B**).

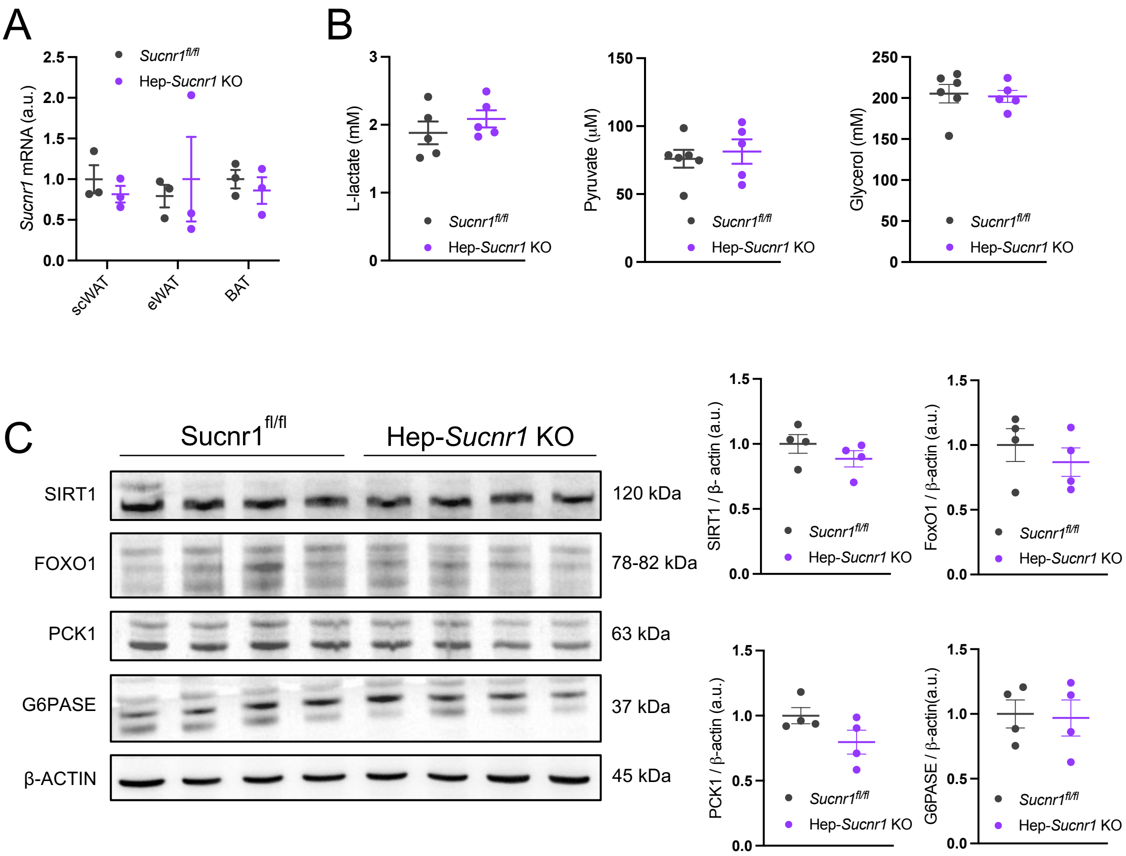

Fig. S3. Hep-*Sucnr1* KO mice do not show changes in *Sucnr1* expression in adipose tissue depots, alterations in plasma lactate, pyruvate, and glycerol, nor dysregulated gluconeogenic markers expression in primary hepatocytes. RT-qPCR of *Sucnr1* in subcutaneous (scWAT), epididymal (eWAT) and brown adipose tissue (BAT) from Hep-*Sucnr1* KO mice compared to *Sucnr1*$^{fl/fl}$ mice (*n*=3-3) (**A**). Plasma L-lactate (*n*=5-5), pyruvate (*n*=6-5), and glycerol (*n*=6-5) in Hep-*Sucnr1* KO mice compared to *Sucnr1*$^{fl/fl}$ mice after an overnight fasting (**B**). Immunoblot of gluconeogenic markers in hepatocytes isolated from *Sucnr1*$^{fl/fl}$ and Hep-*Sucnr1* KO mice (*n*=4-4) (**C**). Results are presented as mean ± SEM (two-tailed unpaired *t* test).

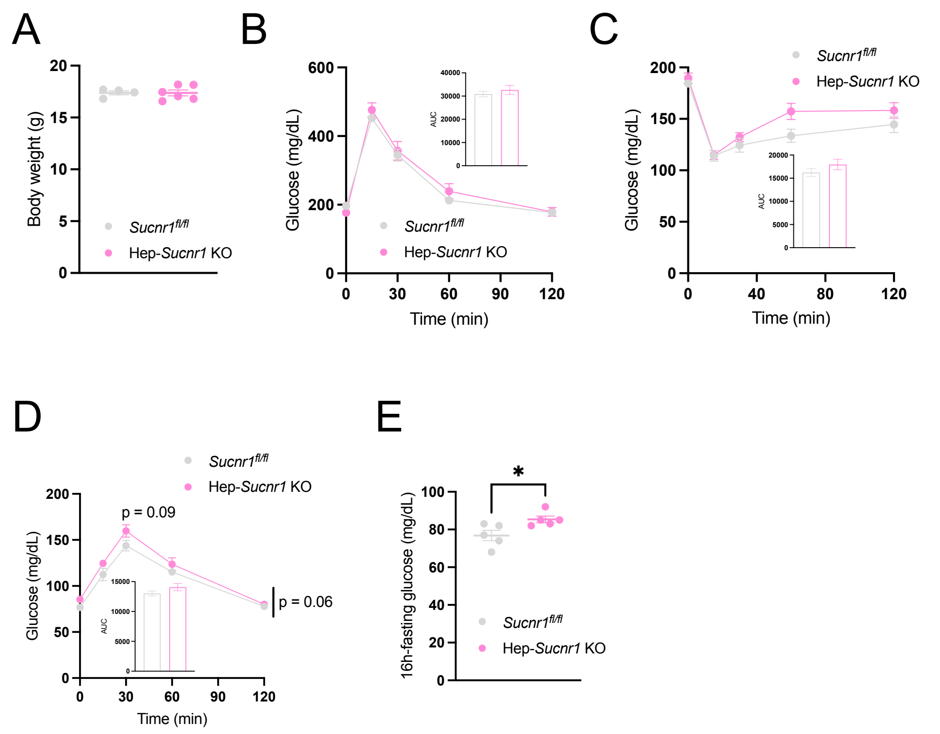

Fig. S4. Hep-*Sucnr1* KO female mice show a similar phenotype to male mice. *Sucnr1*$^{fl/fl}$ and Hep-*Sucnr1* KO female mice were studied at 10 weeks of age. Body weight (*n*=4-6) (**A**). Glucose tolerance test and area under the curve (*n*=4-4) (**B**). Insulin tolerance test and area under the curve (*n*=6-10) (**C**). Pyruvate tolerance test and area under the curve (*n*=5-5) (**D**). Plasma glucose after an overnight fasting (*n*=5-5) (**E**). Results are presented as ± SEM * p<0.05 (two-tailed unpaired *t* test for bar graphs and scatter dot plots; two-way ANOVA for *x*-*y* graphs).

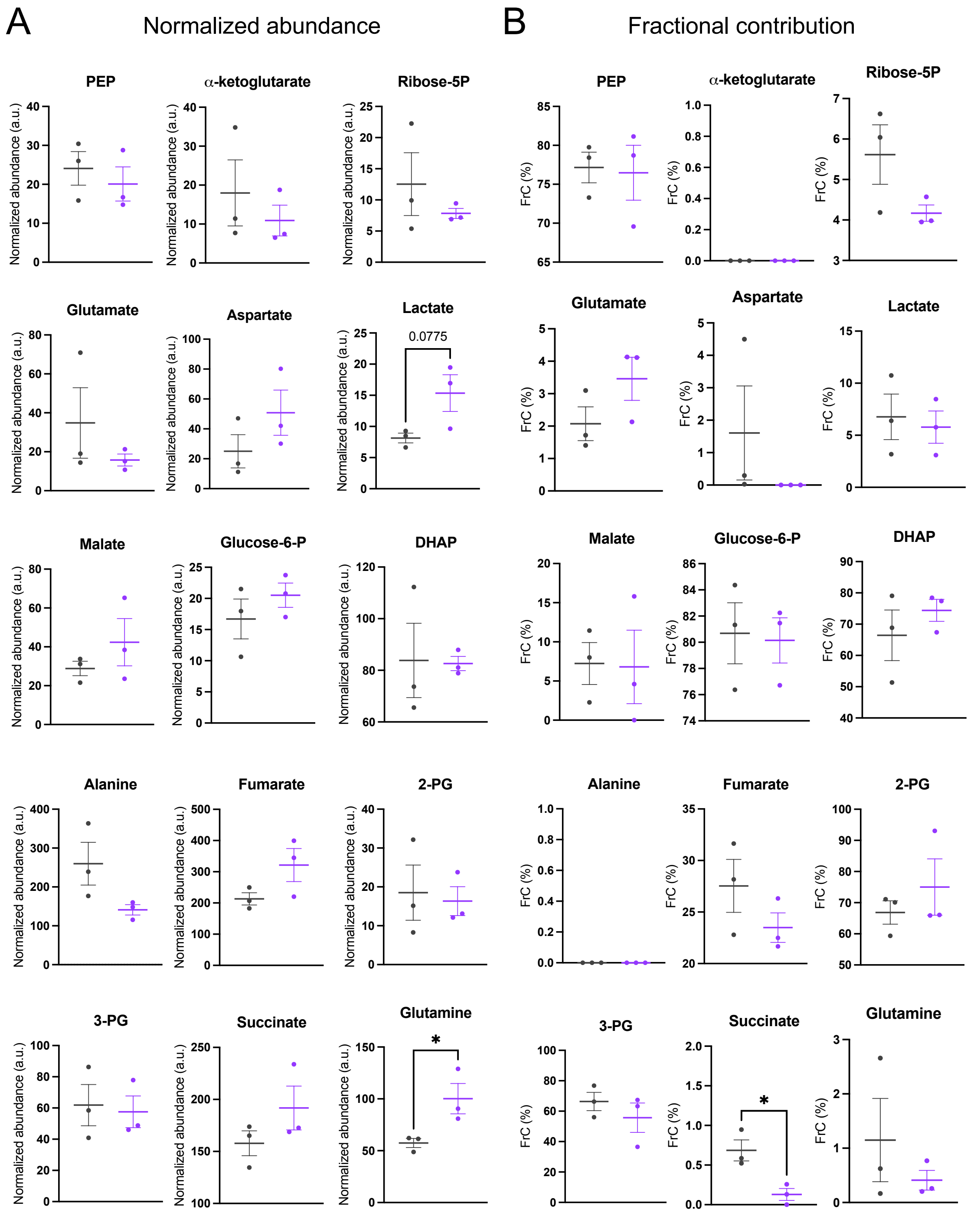

Fig. S5. Isotope-based flux analysis in hepatocytes from *Sucnr1*$^{fl/fl}$ and Hep-*Sucnr1* KO mice treated with ^{13}C-glucose. Normalized abundance (**A**). Fractional contribution (**B**). Results are presented as mean ± SEM (n=3-3). *p<0.05 (two-tailed unpaired t test or Mann-Whitney test).

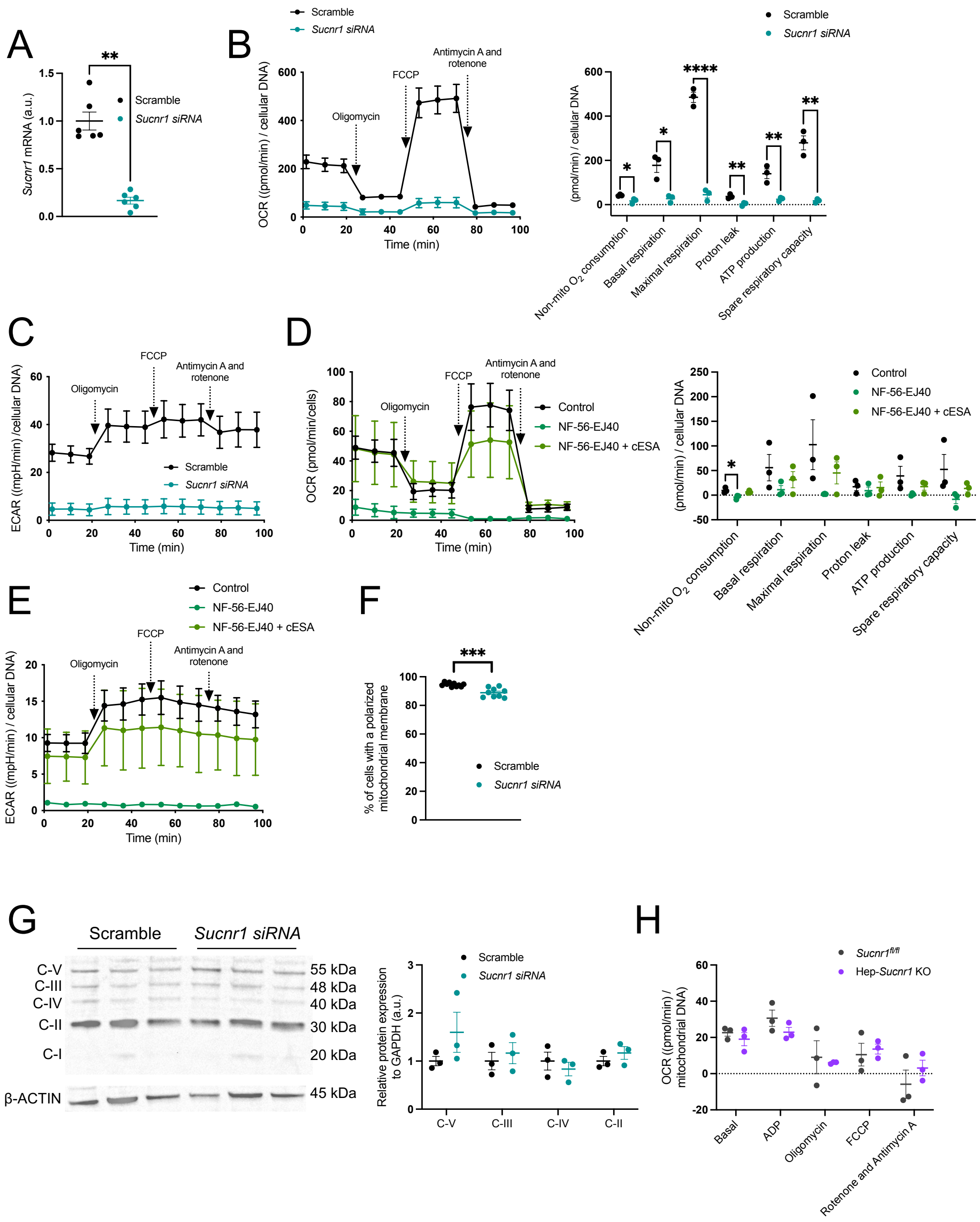

A
Sucnr1 mRNA (a.u.)
Scramble
Sucnr1 siRNA
**
B
Scramble
Sucnr1 siRNA
OCR ((pmol/min) / cellular DNA)
Oligomycin
FCCP
Antimycin A and rotenone
Time (min)
(pmol/min) / cellular DNA
Non-mito O2 consumption
Basal respiration
Maximal respiration
Proton leak
ATP production
Spare respiratory capacity
C
ECAR ((mpH/min) /cellular DNA)
Oligomycin
FCCP
Antimycin A and rotenone
Scramble
Sucnr1 siRNA
Time (min)
D
OCR (pmol/min/cells)
Oligomycin
FCCP
Antimycin A and rotenone
Control
NF-56-EJ40
NF-56-EJ40 + cESA
Time (min)
(pmol/min) / cellular DNA
Non-mito O2 consumption
Basal respiration
Maximal respiration
Proton leak
ATP production
Spare respiratory capacity
E
Control
NF-56-EJ40
NF-56-EJ40 + cESA
ECAR ((mpH/min) / cellular DNA)
Oligomycin
FCCP
Antimycin A and rotenone
Time (min)
F
% of cells with a polarized mitochondrial membrane
***
Scramble
Sucnr1 siRNA
G
Scramble
Sucnr1 siRNA
C-V
C-III
C-IV
C-II
C-I
β-ACTIN
55 kDa
48 kDa
40 kDa
30 kDa
20 kDa
45 kDa
Relative protein expression to GAPDH (a.u.)
Scramble
Sucnr1 siRNA
C-V
C-III
C-IV
C-II
H
Sucnr1fl/fl
Hep-Sucnr1 KO
OCR ((pmol/min) / mitochondrial DNA)
Basal
ADP
Oligomycin
FCCP
Rotenone and Antimycin A

Fig. S6. *Sucnr1* knockdown and blocking in hepatocytes alters mitochondrial function. *Sucnr1* mRNA expression levels in response to 24h of *Sucnr1* siRNA treatment in AML12 cells (*n*=6-6) (**A**). Oxygen consumption rate (OCR), non-mitochondrial O_2 production, basal respiration, maximal respiration, proton leak, ATP production, and spare respiratory capacity in AML12 in response to *Sucnr1* siRNA treatment for 24h (*n*=3-3) (**B**). Extracellular acidification rate (ECAR) in response to *Sucnr1* siRNA treatment for 24h (*n*=3-3) (**C**). OCR, non-mitochondrial O_2 production, basal respiration, maximal respiration, proton leak, ATP production, and spare respiratory capacity in response to SUCNR1 antagonist (5h) or SUCNR1 antagonist (5h) + cESA (3h) in THLE-2 (*n*=3-3) (**D**). ECAR in response to SUCNR1 antagonist or SUCNR1 antagonist (5h) + cESA (3h) in THLE-2 (*n*=3-3) (**E**). Mitochondrial membrane polarization in AML12 cells in response to 24h of *Sucnr1* siRNA treatment (*n*=3-3) (**F**). Immunoblot of total OXPHOS antibody cocktail in AML12 cells in response to 24h of *Sucnr1* siRNA treatment (*n*=3-3) (**G**). Basal, ADP-induced, oligomycin-inhibited, FCCP-induced and Rotenone/Antimycin A-inhibited OCR in response to CI-substrates malate and pyruvate in isolated mitochondria from *Sucnr1*$^{fl/fl}$ and Hep-*Sucnr1* KO mice (*n*=3-3) (**H**). Numeric results are presented as mean ± SEM and statistical significance studied by two-tailed unpaired *t* test; *$p<0.05$; **$p<0.01$; ***$p<0.001$; ****$p<0.0001$.

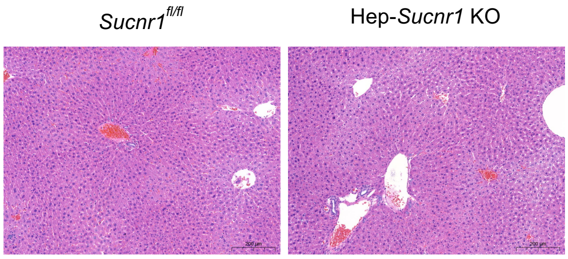

Fig. S7. *Sucnr1* deficiency in hepatocytes does not affect hepatic histology. Liver H&E staining of *Sucnr1*$^{fl/fl}$ and Hep-*Sucnr1* KO mice.

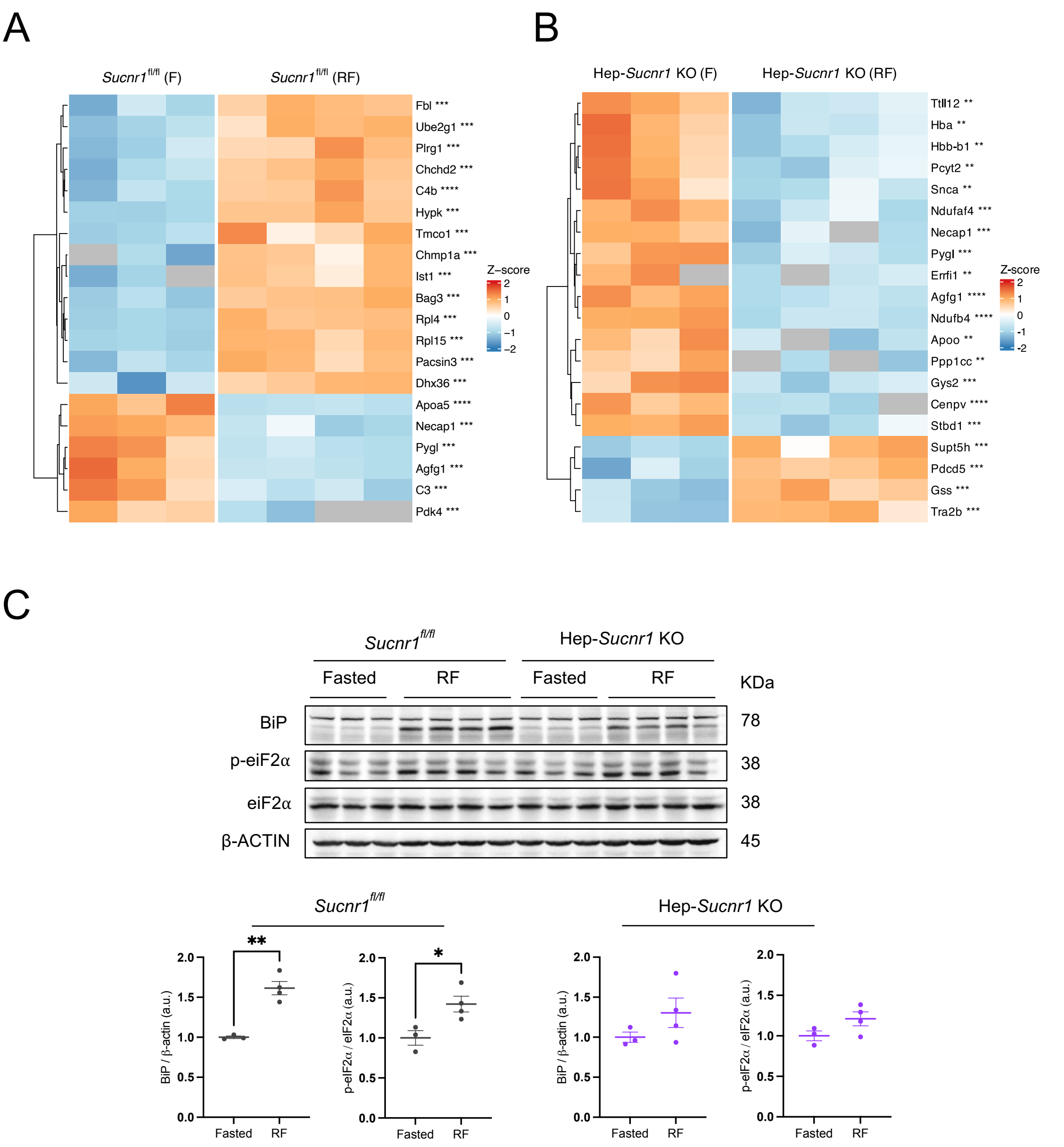

Fig. S8. Refeeding induces a distinct and attenuated hepatic response in Hep-*Sucnr1* KO mice. Heatmap of the top 20 most regulated proteins in refeeding (RF) vs fasting (F) in the livers of *Sucnr1*$^{fl/fl}$ mice (*n*=4-3) (**A**) and Hep-*Sucnr1* KO mice (*n*=3-4) (**B**). Immunoblot analysis of ER stress markers binding immunoglobulin protein (BiP), phosphorylated eukaryotic translation initiation factor 2 alpha (p-eIF2α), and total eIF2α in response to RF in *Sucnr1*$^{fl/fl}$ (*n*=3-4) and Hep-*Sucnr1* KO mice (*n*=3-4), with densitometric quantification of BiP and the p-eIF2α/eIF2α ratio normalized to β-actin (**C**). Results are presented normalized as Z-score (A, B) or as mean ± SEM (C), and statistical significance studied by limma's empirical Bayes moderated *t* statistics (A, B) or two-tailed unpaired *t* test (C).

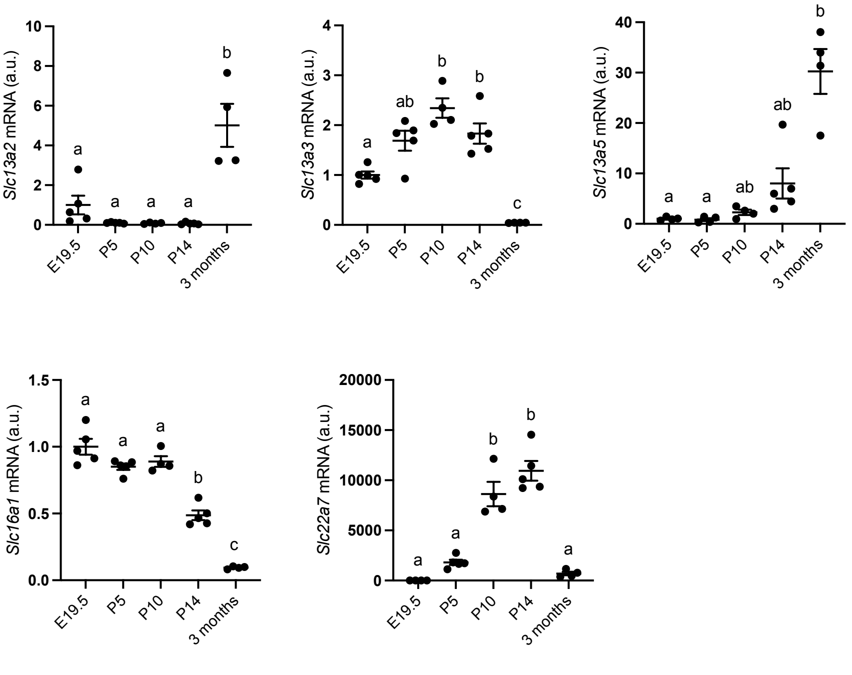

Fig. S9. Succinate transporters are modulated during early postnatal development. Gene expression analysis of succinate transporters at E19.5, P5, P10, P14, and 3 months of age (*n*=4-5). Results are presented as mean ± SEM, and statistical significance studied by one-way ANOVA plus Tukey's multiple comparisons test or Kruskal-Wallis plus Dunn's multiple comparisons test. Significantly different groups (p<0.05) are shown with different letters, while non-significant comparisons show the same letter.

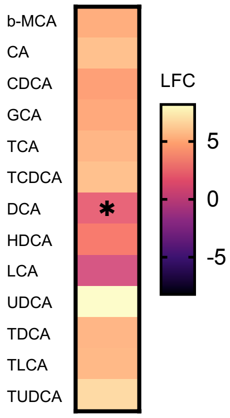

Fig. S10. Hep-*Sucnr1* KO mice show increased bile acids in circulation. Bile acids determination in plasma in fasting conditions. Data are presented as $\log_2$ fold change (LFC) of the mean of Hep-*Sucnr1* KO mice vs. *Sucnr1*$^{fl/fl}$ mice (*n*=3-3). **p*<0.05 (two-tailed unpaired *t* test). B-MCA, b-Muricholic acid; CA, cholic acid; CDCA, chenodeoxycholic acid; GCA, glycocholic acid; TCA, taurocholic acid; TCDCA, taurochenodeoxycholic acid; DCA, deoxycholic acid; HDCA, hyodeoxycholic acid; LCA, lithocholic acid; UDCA, ursodeoxycholic acid; TDCA, taurodeoxycholic acid; TLCA, taurolithocholic acid; TUDCA, tauroursodeoxycholic acid.

Table S1. Summary of significant proteins per comparison in the proteomics study

Significant proteins per comparison

	p values		Adjusted *p* values	
Comparison	*p* value <0.05	*p* value <0.01	Adj *p* value <0.1	Adj *p* value <0.05
**Hep-*Sucnr1* KO F vs *Sucnr1*$^{fl/fl}$ F**	147	35	1	0
***Sucnr1*$^{fl/fl}$ RF vs *Sucnr1*$^{fl/fl}$ F**	475	146	22	0
**Hep-*Sucnr1* KO F vs Hep-*Sucnr1* KO RF**	195	52	3	1

Abbreviations: F, fasted; RF, refed

Table S2. Pericentral, periportal, and Wnt pathway gene sets

Pericentral genes								
Acot1	*Ces2d-ps*	*Cyp2a5*	*Cyp2e1*	*Glul*	*Hpgd*	*Oat*	*Rnf43*	*Slc22a1*
Adh6-ps1	*Ces2h*	*Cyp2c29*	*Cyp2g1*	*Gsta3*	*Lcn2*	*Orm2*	*Saa2*	*Slc22a3*
Ahr	*Cib2*	*Cyp2c37*	*Cyp3a59*	*Gstm1*	*Lect2*	*Pcp4l1*	*Saa3*	*Sntb1*
Akr1b7	*Cxcl1*	*Cyp2c38*	*Cyp7a1*	*Gstm2*	*Lgr5*	*Pdk4*	*Scd2*	*Sult1b1*
Akr1c20	*Cxcl9*	*Cyp2c39*	*Endod1*	*Gstm3*	*Lhpp*	*Pfkfb1*	*Slc13a3*	*Sult1e1*
Axin2	*Cyp1a2*	*Cyp2c50*	*Fam89a*	*Gstm6*	*Ncald*	*Plbd1*	*Slc16a10*	*Tbx3*
Blvrb	*Cyp27a1*	*Cyp2c54*	*Gbp11*	*Gulo*	*Ndrg1*	*Por*	*Slc16a7*	*Ube2e2*
Ces2b	*Cyp2a22*	*Cyp2c55*	*Gck*	*Hip1r*	*Npr2*	*Rec8*	*Slc1a2*	*Ugt2b37*
Ces2c	*Cyp2a4*	*Cyp2d40*	*Gda*	*Hopx*	*Nr1i3*	*Rhbg*	*Slc1a4*	*Vnn1*

Periportal genes						
Acly	*Bdh2*	*Cryl1*	*Cyp4a12a*	*Hsd17b6*	*Scnn1a*	*Slc7a2*
Alb	*Cd5l*	*Ctnnbip1*	*Enho*	*Igfbp2*	*Sds*	*Sult5a1*
Adh1b1	*Cdh1*	*Ctsc*	*Gas2*	*Mafb*	*Sdsl*	*Tbc1d30*
As3mt	*Celsr1*	*Cux2*	*Gls2*	*Mfsd2a*	*Serpina12*	*Ugt2b38*
Asl	*Clec2h*	*Cyp17a1*	*Gpc1*	*Mmd2*	*Serpina4-ps1*	
Aspg	*Clec4f*	*Cyp2f2*	*Gsta2*	*Mup20*	*Setd7*	
Ass1	*Cobl*	*Cyp2u1*	*Hes1*	*Pck1*	*Slc13a2*	

Wnt pathway genes							
Ahr	*Fzd3*	*Glul*	*Lgr5*	*Lrp2bp*	*Reck*	*Sdc2*	*Wnt2*
Ascl2	*Fzd4*	*Gpc1*	*Lrp1*	*Lrp3*	*Rnf43*	*Sdc3*	*Wnt4*
Axin2	*Fzd5*	*Gpc3*	*Lrp10*	*Lrp4*	*Ror1*	*Sdc4*	*Wnt5a*
Ccnd1	*Fzd6*	*Gpc4*	*Lrp11*	*Lrp5*	*Rspo1*	*Sfr1*	*Wnt5b*
Cd44	*Fzd7*	*Igfbp4*	*Lrp12*	*Lrp6*	*Rspo3*	*Sfrp1*	*Wnt9b*
Dkk3	*Fzd8*	*Kremen1*	*Lrp1b*	*Myc*	*Ryk*	*Sfrp5*	*Znrf3*
Fzd1	*Fzd9*	*Lgr4*	*Lrp2*	*Notum*	*Sdc1*	*Sox9*	

Table S3. Gene set enrichment analysis specifications

DESCRIPTION	SET SIZE	ENRICHMENT SCORE	NES	*P* VALUE	*P* ADJUST	*Q* VALUE	RANK	LEADING EDGE
**PERICENTRAL**	80	0.5383977	4.67 3525	1.00e-10	3.00e-10	NA	2630	tags=64%, list=17%, signal=53%
**PERIPORTAL**	45	-0.3357927	- 2.43 8300	4.65e-04	6.97e-04	NA	3602	tags=51%, list=24%, signal=39%
**WNT**	46	-0.2374091	- 1.73 7559	2.05e-02	2.05e-02	NA	6647	tags=65%, list=44%, signal=37%

Abbreviations: NES, normalized enrichment score.

Table S4. m/z, retention time, and number of isotopologues for each metabolite in the isotope-based flux analysis

Metabolite	m/z	Retention time	Number of isotopologues
**Pyruvate**	87.0088	3.78 min	4
**Citrate**	191.0197	4.38 min	7
**Aconitate**	173.0092	3.71 min	7
**α-Ketoglutarate**	145.0142	3.80 min	6
**Succinate**	117.0193	2.95 min	5
**Fumarate**	115.0037	3.92 min	5
**Malate**	133.0142	4.39 min	5
**Phosphoenolpyruvate**	166.9751	4.20 min	4
**Glucose**	179.0567	3.76 min	7
**Glucose-6-phosphate**	259.0224	4.38 min	7
**Fructose-6-phosphate**	259.0224	4.22 min	7
**Dihydroxyacetone phosphate**	168.9907	4.09 min	4
**Glyceraldehyde-3-phosphate**	168.9907	4.24 min	4
**2-Phosphoglycerate**	184.9857	4.19 min	4
**3-Phosphoglycerate**	184.9857	4.36 min	4
**Ribose**	149.0455	3.70 min	6
**Ribose-5-phosphate**	229.0119	4.21 min	6
**6-Phosphogluconate**	275.0179	4.53 min	7
**Alanine**	88.0404	4.03 min	4
**Valine**	116.0717	3.68 min	6
**Glycine**	74.0248	4.32 min	3
**Serine**	104.0353	4.33 min	4
**Aspartic acid**	132.0302	4.23 min	5
**Lactate**	89.0244	2.60 min	4
**Glutamine**	145.0619	4.28 min	6
**Glutamate**	146.0459	4.19 min	6
**d_3-Leucine**	133.1062	3.33 min	Internal standard

Table S5. TaqMan probes for mRNA expression

Gene	Description	TaqMan probe
B2m	Beta-2-microglobulin	Mm00437762_m1
Slc13a2	Solute carrier family 13, member 2	Mm00452095_m1
Slc13a3	Solute carrier family 13, member 3	Mm00475289_m1
Slc13a5	Solute carrier family 13, member 5	Mm01334459_m1
Slc16a1	Solute carrier family 16 (monocarboxylic acid transporters), member 1	Mm01306379_m1
Slc22a13	Solute carrier family 22 (organic anion transporter), member 13	Mm07299946_g1
Slc22a7	Solute carrier family 22 (organic anion transporter), member 7	Mm00468672_m1
Sucnr1	Succinate receptor 1	Mm02620543_s1

Table S6. Antibodies for Western Blot

Antibody	Dilution	Host species	Reference and source
Anti-ATF4 antibody	1:1000	Rabbit	#ab216839, Abcam
Anti-beta-Actin antibody	1:2000	Mouse	#A1978, Sigma-Aldrich
Anti-Fox01 (C29H4)	1:1000	Rabbit	#2880, Cell Signaling Technology
Anti-GADD 153 (CHOP) polyclonal rabbit antibody	1:1000	Rabbit	#sc-575, Santa Cruz Biotechnology
Anti-G-6-Pase	1:1000	Rabbit	#ab83690, Abcam
Anti-mouse IgG Secondary Antibody, HPR	1:2000	Goat	#62-6520, Invitrogen
Anti-PCK1 (D12F5)	1:1000	Rabbit	#12940, Cell Signaling Technology
Anti-phospho-mTOR (Ser2448)	1:1000	Rabbit	#2971, Cell Signaling Technology
Anti-rabbit IgG HRP linked whole antibody	1:2000	Donkey	#NA934V, ECL
Anti-SirT1 (1F3)	1:1000	Mouse	#8469, Cell Signaling TEchnology
ATF6 Antibody	1:1000	Rabbit	#NBP1-40256SS, Novus Biologicals
BiP (C50B12) Rabbit Monoclonal Antibody	1:1000	Rabbit	#3177, Cell Signaling Technology
eIF2 alpha Antibody	1:1000	Rabbit	#9722, Cell Signaling Technology
GAPDH Loading Control Antibody (GA1R)	1:1000	Mouse	#MA5-15738, Thermo Scientific
mTOR Antibody	1:1000	Rabbit	#2972, Cell Signaling Technology
OxPhos Rodent WB Antibody Cocktail	1:250	Mouse	#45-8099, Invitrogen
Phospho-eIF2 alpha (Ser51) Antibody	1:1000	Rabbit	#9721, Cell Signaling Technology

Supplemental auxiliary files (separate files)

Excel tables

Data S1. Raw data for Fig. 1

Data S2. Raw data for Fig. 2

Data S3. Raw data for Fig. 3

Data S4. Raw data for Fig. 4

Data S5. Raw data for Fig. 5

Data S6. Raw data for Fig. 6

Data S7. Raw data for Fig. S1

Data S8. Raw data for Fig. S3

Data S9. Raw data for Fig. S4

Data S10. Raw data for Fig. S5

Data S11. Raw data for Fig. S6

Data S12. Raw data for Fig. S8

Data S13. Raw data for Fig. S9

Data S14. Raw data for Fig. S10

Data S15. Proteomics raw data
